# Trauma and Trust: How War Exposure Shapes Social and Institutional Trust Among Refugees

**DOI:** 10.3389/fpsyg.2022.786838

**Published:** 2022-08-16

**Authors:** Jonathan Hall, Katharina Werner

**Affiliations:** ^1^Department of Peace and Conflict Research, Uppsala University, Uppsala, Sweden; ^2^School of Business, Economics and Information Systems, University of Passau, Passau, Germany

**Keywords:** institutional (dis)trust, social trust, refugee, conflict exposure, trauma, post-traumatic growth, post-traumatic stress, Syria and Iraq

## Abstract

The brutal wars in Iraq, Syria and now Ukraine have caused a massive influx of refugees to Europe. Turkey alone has received more than 4.8 million refugees. An important precondition for their economic and social incorporation is trust: refugees need to trust the citizens as well as the state and the justice system to find their place in the host country. Yet refugees’ propensity to trust may be affected by cultural differences between their home and host countries, their personal conflict exposure and the experiences they had on the run. This study investigates how individual differences in exposure to armed conflict and institutional breakdown shape two types of trust among refugees: Generalized social trust and trust in the institutions of the settlement country. We survey a large and diverse sample of refugees from Syria and Iraq living in Turkish communities and deploy well-established measures of conflict exposure, posttraumatic stress, and posttraumatic growth. We find that higher degrees of conflict exposure are positively related to social trust, and to trust in courts and the police. These positive findings are largely driven by refugees who had very personal and emotionally powerful experiences. The psychological mechanism of posttraumatic growth cannot explain these findings, however, suggesting positive experiences of cooperation in the midst of war and displacement are potentially a better explanation for this finding than positive psychological changes resulting from trauma. At the same time, conflict exposure is negatively related to trust in political institutions. Posttraumatic stress may be the mechanism behind this result. We discuss the implications of these findings for the integration of war refugees—a topic that is tragically of great relevance today.

## Introduction

Following the large influx of refugees in recent years from Syria and Iraq and the current influx from Ukraine, one of the major challenges facing Europe is to encourage their social and economic incorporation. Quick and sustained incorporation can enable host countries to benefit from refugees. For example, refugees can help avoid a demographic crisis and ease the increasing pressure on the social system caused by low birth rates. At the same time, failure to incorporate refugees may threaten social cohesion and cooperation in host countries and induce hostility between natives and refugees, or even amongst natives. Besides these social threats, such failure may harm the host country’s economy, for example by increasing unemployment rates and the resulting transfer payments (Gathmann and Keller, 2018).

Social capital in general, and trust in particular, have been highlighted as essential for peaceful and stable societies (Putnam et al., 1993; Fukuyama, 1995; Knack and Keefer, 1997; La Porta et al., 1997; Newton, 2001; Uslaner, 2002; Hynes, 2009; Bjørnskov, 2012). Broadly speaking, trust is the belief in the reliability of someone or something. Trust enables people to cooperate (Coleman, 1988; Gambetta, 1988; Buchan et al., 2002; Uslaner, 2002; Stanley, 2003; Larsen, 2014), because most people only cooperate if they expect that others will do so as well. It allows people to resolve disputes and thus avoid conflict (Dent, 2005). Social capital and trust are also regarded as fundamental prerequisites for collective action and civic culture, because they are considered the “glue” that holds societies together (Axelrod and Hamilton, 1981; Rothstein, 2005). They thus provide the basis for functioning markets and economic growth (e.g., Arrow, 1972; Knack and Keefer, 1997; Zak and Knack, 2001; Bjørnskov, 2009).

Trust is particularly important in light of immigrants arriving in a foreign culture, because cooperation is often more pronounced within groups than between groups (e.g., Goette et al., 2006; Ruffle and Sosis, 2006; Masuda, 2012; Balliet et al., 2014; El-Bialy et al., 2022). Moreover, cooperation is likely to be higher between groups that perceive each other as more similar than between very distinct groups (Froehlich et al., 2021). The incorporation of refugees can thus be deemed a particularly challenging act of intercultural cooperation that requires efforts from both the refugees and the host community. While shared values tend to increase the willingness to cooperate, strong cultural differences may, on the other hand, increase the perceived risk of misunderstanding and defection. To foster cohesion in a diverse society—for example in a country that has accepted refugees from various other countries—shared expectations of each other’s trustworthiness are thus vital. Therefore, trust is often highlighted as the key to the future of refugees in the host society (Hynes, 2009; Strang and Ager, 2010; Strang and Quinn, 2021; El-Bialy et al., 2022). When refugees trust members of the host society and vice versa, refugees are better able to build up their social networks and contribute to the host society. At the same time, even in the presence of cultural differences, trust in institutions may alleviate the problem of potential misunderstandings and make people confident that they can rely on each other.

This shows that two forms of trust are important for successful adaptation: Generalized social trust and institutional trust. We have to point out here that—in contrast to other migrants—war refugees usually do not have the choice to migrate to a country they deem particularly attractive. As long as the costs of staying in their home country are sufficiently high, they may even be willing to live in a country they do not find trustworthy. Hence, trust toward individuals and institutions in the host country cannot be taken for granted. The refugees’ level of generalized social trust may reflect their perception of sharing similar values with people in their new environment (Bouckaert and Dhaene, 2004; Henrich et al., 2005; Bigoni et al., 2016; Karaja and Rubin, 2021) and is thus an indicator of their willingness to interact and cooperate with them (e.g., Nickerson et al., 2019). The refugees’ levels of institutional trust in the host country can indicate how much they embrace the host country’s culture, norms, and other country-specific factors, and how protected they feel by the country’s institutions. It is thus, among other things, a good indicator of how much refugees are willing to participate in economic interactions and contribute to society.

While both sides benefit from mutual trust, trust always involves vulnerability and the risk of disappointment if other persons do not behave as expected (Gambetta, 1988; Williamson, 1993; Levi and Stoker, 2000; Cox, 2004, 2007; Johnson and Mislin, 2011). Apart from cultural similarities or differences, the (positive or negative) experiences people have in their lives are particularly likely to affect their beliefs and perception of such a risk (Glanville and Paxton, 2007; Uslaner, 2008; Dinesen and Bekkers, 2017). Hence, the experiences refugees had on the run (BenEzer and Zetter, 2015; Hynes, 2017; Essex et al., 2021), their perception of being in a safe environment, and their prospects for receiving asylum (Jakobsen et al., 2017) may affect their trust levels. The impact of the context from which the refugees came may interact with the context in which they live now (Ní Raghallaigh, 2014). Even more importantly, in contrast to most other people, refugees share particularly salient and potentially traumatic experiences that forced them to leave their home country. People’s experiences of violence, war, and death (Hall and Kahn, 2020; Hall et al., 2021), as well as the varied mental health trajectories that unfold in response to such experiences (Canevello et al., 2021), may plausibly have had a lasting impact on their perceptions and shape the lens through which they view the people they meet in their new environment. Similarly, the refugees’ experiences with institutions in their home country in times of violence and war may shape the lens through which they view institutions in the host country.

This study investigates how refugees’ exposure to violence and conflict affects their generalized social trust and institutional trust in the host country. We thus bring together the literature on the determinants of immigrant trust with the literature on the political and social effects of war violence. In contrast to the existing migration literature, we do not examine cultural and institutional factors as determinants of trust among immigrants. Instead, we focus on individual differences in war exposure in a sample of forcibly displaced people. We survey a large sample of Iraqi and Syrian refugees in Turkey, using well-established measures of conflict exposure and the subsequent psychological trajectories of post-traumatic stress (PTSD) and post-traumatic growth (PTG). Trauma may be an important driver of potential effects of conflict exposure on trust (Mooren and Kleber, 2001). Data were gathered amid Turkey’s 2016 political turmoil, which is likely to make the experiences from their war-torn home countries more salient. We exploit the tragic fact that the violence in Syria and Iraq was largely indiscriminate—including practices such as barrel bombing, indiscriminate shelling, and even chemical attacks—and can thus plausibly be deemed as-if random (Human Rights Watch, 2018; Fabbe et al., 2019). To the best of our knowledge, this is the first study to investigate the relationship between trust and conflict exposure among refugees using such a large sample and well-established survey measures. Another innovation in this study is the disaggregation of different types of conflict exposure. Based on the psychological literature, we hypothesize and show that the propensity to trust depends on individual differences related to the experience of armed conflict in one’s home country—in particular, on the type of war exposure and posttraumatic stress refugees experienced.

## Background

### Literature Review

Generalized social trust is commonly defined as a general willingness to trust strangers, i.e., people we do not have any information about (Uslaner, 2002; Bahry et al., 2005). Institutional trust, on the other hand, rests upon people’s perceptions of how well the social and organizational environment in a society operates (e.g., Miller, 1974; Williamson, 1993; Halapuu et al., 2013). Institutional trust can help to overcome the problems of collective action (Fukuyama, 1995; Putnam, 2000), and it has been shown that institutional trust helps improve native people’s attitudes toward immigrants and minorities (Espenshade and Hempstead, 1996; Husfeldt, 2006; Halapuu et al., 2013). Likewise, institutional trust could also shape immigrants’ willingness to cooperate with people and organizations in the host country, which could have an impact on their propensity to participate in social interactions and the labor market. Some authors point out that there may be strong correlations between social and institutional trust and that one of these forms of trust can serve the other (Rothstein and Uslaner, 2005; Hynes, 2009; Strang and Ager, 2010). In particular, trust in state institutions that govern individuals’ interactions, such as the judiciary and the police, may be an important prerequisite for social trust (Levi, 1996; Rothstein and Stolle, 2008; Dinesen and Bekkers, 2017). To explain levels of social and institutional trust among immigrants, the previous literature focuses on two factors: (1) immigrant cultural background, and (2) settlement country institutions. For example, it has plausibly been argued that trust levels relate to the conditions of reception in the host country and the perception of being safe, both in physical and economic terms (e.g., Ager and Strang, 2008; Strang and Ager, 2010; Steel et al., 2011; Hainmueller et al., 2015; Slewa-Younan et al., 2015; Jakobsen et al., 2017; Gathmann and Keller, 2018; Felfe et al., 2021). Other studies show that many new immigrants exhibit even higher levels of institutional trust than the native population. Yet, these differences attenuate over time and across generations (Maxwell, 2010; Adman and Strömblad, 2015). A prominent explanation is that experiences of poorer governance in the countries of origin result in high trust and satisfaction in settlement country institutions. However, as immigrants have new experiences in the host country and become more similar to natives (or, alternatively, more aware of discrimination), their trust in political institutions declines.

But a particularly powerful experience refugees may have had is often ignored in the migration literature. This experience is their exposure to conflict and violence. Such exposure may comprise various experiences and different kinds of traumatic stresses. A strong degree of conflict exposure may constitute a major shock and some of these experiences may have a significant impact on immigrants’ social and political attitudes. Conflict exposure often comprises two aspects: personal trauma or other (negative) social experiences on the one hand, and institutional breakdown or violence perpetrated by state actors on the other. Various forms of conflict exposure are thus likely to impact both social trust and institutional trust and individual differences in such exposure may help to explain differences in trust among refugees.

In the literature on social trust, there are diverging opinions on how exposure to violent conflicts affects social trust.^1^ One view finds conflict exposure reduces social trust. During a conflict, people usually experience a lot of destruction and may lose their social networks due to displacement and death. People may perceive their life to be in danger. They may be physically threatened or injured or witness others being killed (Hayes and McAllister, 2001, p. 908; Bertrand, 2002, p. 75; Spyer, 2002, p. 25). They may not know whom to trust because they experience people turning against their own neighbors or relatives. Such experiences may reduce their sense of security and general faith in humans, resulting in heightened threat perceptions and distrust of other people (Posen, 1993; Colletta and Cullen, 2000; Mooren and Kleber, 2001; Ingelaere, 2009; Whitt, 2010; Cassar et al., 2013; De Luca and Verpoorten, 2015b). Not only personal experiences but even mere indirect exposure, for example through reports by friends or the media, can induce similar threat perceptions and distrust (Dixon et al., 1993; Slone, 2000; Thabet et al., 2002; Engel and Ibáñez, 2007; Schmid et al., 2008; Turnip et al., 2010; Schmid and Muldoon, 2013). For example, Werner and Skali (2021) show in an experimental and survey-based study that people with indirect exposure to conflict exhibit significantly lower levels of trust in all people, irrespective of group membership.

On the other hand, a relatively new line of literature argues that conflict exposure may even have positive effects on social capital and trust (Lyons et al., 1998; Bellows and Miguel, 2009; Blattman, 2009; Voors et al., 2012; Gilligan et al., 2014; De Luca and Verpoorten, 2015a; see Bauer et al., 2016 for a meta-analysis). The psychological literature provides a possible explanation for this phenomenon: The concept of posttraumatic growth claims that people may undergo positive changes through struggling with traumatic experiences. People may find new meaning and purpose in life and develop stronger moral convictions as a result of what happened (Tedeschi and Calhoun, 1996, 2004; Powell et al., 2003; Staub and Vollhardt, 2008; Ramos and Leal, 2013; Williamson, 2014; Bhat et al., 2015). An alternative explanation relates to positive experiences people may have had during the conflict. Despite all the suffering, they may have experienced a lot of mutual support. For example, people may have cooperated to defend themselves or even risked their lives to defend others (Tucker and Ferson, 2008, p. 114). People may have jointly reconstructed destroyed buildings (Barron et al., 2010). They may have experienced collective coping and the warm hug of people giving comfort to them when they lamented the death of a relative (Pennebaker and Harber, 1993; Lyons et al., 1998; Gilligan et al., 2014, pp. 615–616). People who were injured may have received care by others. Other people may have obtained shelter after they had to leave their own house. All these conflict experiences are likely to have a strong impact on refugees’ trust in other people and state institutions.

The existing literature on the integration of refugees so far has often focused on the attitudes of host country citizens toward refugees (e.g., Lazarev and Sharma, 2017; Adida et al., 2018; Böhm et al., 2018; Hassan et al., 2019; Fiedler et al., 2021). Studies with a focus on the refugees’ behavior have examined social preferences like altruism (El-Bialy et al., 2020), risk preferences (El-Bialy et al., 2020), honesty (El-Bialy et al., 2021a), punishment behavior (El-Bialy et al., 2021b) and cooperation (El-Bialy et al., 2022). Very few studies investigate trust. In a recent systematic literature review, Essex et al. (2021) identify 24 studies that explore trust among refugees. Out of these, only six are quantitative studies. Essex et al. (2021) highlight the need to explore the impact of context, such as post-migration structures, and pre-migration experiences, on refugees’ trust. The only study in their meta-analysis that takes pre-migration experiences into account is by Mooren and Kleber (2001), who show that Bosnian refugees express lower trust both in other people and in authorities, compared to Dutch students. They elicit measures of coping and briefly discuss their relationship to trust among other outcomes but do not investigate the relationship between exposure to different types of potentially traumatic wartime events and social and institutional trust. Furthermore, their sample of refugees is quite small. This is where our study contributes to the literature. In line with the conclusion by Essex et al. (2021), we set out to examine how generalized social trust and institutional trust depend on personal conflict exposure in a large and diverse sample of Syrian and Iraqi refugees currently residing in Turkey.

### Brief Summary of the Conflicts in Syria and Iraq

#### Syria

The Syrian conflict is regarded as one of the most brutal sectarian conflicts in recent memory. Yet, the conflict was not always centered on religion. In the beginning, the sources of tensions were a growing dissatisfaction with Bashar al-Assad’s regime, Assad’s neglect of rural regions, growing unemployment and a widening of the gap between rich and poor. Corruption and the arbitrariness of security forces further increased dissatisfaction in the population (Droz-Vincent, 2014; McHugo, 2014). Peaceful demonstrations started in the Arab Spring of 2011. The security forces responded by arresting, torturing, and shooting demonstrators. These brutal reactions urged many more people to take to the streets and soon caused a downward spiral of violence (Malantowicz, 2013; Droz-Vincent, 2014; McHugo, 2014). Over time, identity politics that were primarily related to religion became increasingly important. The Assad regime quickly turned to sectarianism to mobilize political support and claimed to be protecting minority groups against the Sunni majority. In a region where religion was a primary marker of identity, it did not take long until toxic sectarianism started to spread. In the time that followed, the perception rose that Alawites were indiscriminately supporting the regime. Hezbollah fighters and Shia volunteers (mainly from Iran and Iraq) started to support the Assad regime. At the same time, fighters from fellow Sunni countries backed the rebels. This increasingly turned the political conflict into a war in which Sunni Muslims opposed Shia Muslims and Alawites (Wimmen and Asseburg, 2012; Malantowicz, 2013; Droz-Vincent, 2014; Jenkins, 2014). The increased use of heavy weapons turned it into one of the most brutal conflicts of our time (World Bank, 2017), characterized by widespread instances of indiscriminate violence. Barrel bombs and chemical weapons were used against civilians in many parts of the country and caused increasingly large waves of forced displacement.^2^

Most of the displaced persons remained in Syria and among those internally displaced persons, the majority even remained in their governorate of origin. The total number of registered Syrian refugees in Lebanon, Jordan, Turkey, Iraq, Egypt and North Africa increased from 21,533 in 2012 to about half a million in 2013, 2.4 million in 2014, 3.8 million in 2015, and 4.9 million in 2016 (United Nations High Commissioner for Refugees)—plus an estimated 0.4**–**1.1 million unregistered persons in these countries. Among these countries, Turkey accounts for the highest number of Syrian refugees (with a total of about 2.8 million in 2016). These numbers do not include an estimated total of more than 800,000 people who fled to Europe. Taken together, as of April 2016, the conflict had caused between 400,000 and 470,000 estimated fatalities. As of January, 2017, with an estimated 6.5 million refugees outside the country and a further 5.7 million internally displaced persons, more than half of Syria’s 2010 population was forcibly displaced (World Bank, 2017). This can be deemed the largest outflow of refugees in recent history, which has resulted in a significant increase in the total number of refugees and asylum seekers worldwide (World Bank, 2018).

#### Iraq

The recent violence in Iraq has its early roots in the economic and social harm caused by Saddam Hussein’s misgovernment and in the US occupation from 2003 onwards. The 2005 elections saw an increasing politicization of religious and ethnic identities. After the elections, the marginalization of the Sunni community in the constitutional process and a gradual but noticeable shift of power toward Shia dominance reinforced ethnic and sectarian fractionalization. A struggle for power between Baghdad and the provinces followed by a rise of insurgent groups exacerbated the situation. The tensions culminated in the outbreak of a sectarian war from 2006 to 2007 (Marr and Al-Marashi, 2017) with high levels of indiscriminate violence (Green and Ward, 2009; Hicks et al., 2011).

The subsequent period of stabilization did not last long. Ethnic and sectarian tensions rose again when discontent with Prime Minister Maliki grew. Conflicting political currents competed in two elections in 2009, and again in 2010, for a new national assembly and government. Maliki’s opponent’s narrow victory, the withdrawal of the US forces in 2011, and a resulting lack of authority further increased the tensions. In 2011, the Arab Spring started in Tunisia and rapidly spread across the region. The government immediately deployed security forces against the demonstrators. Maliki found himself increasingly under criticism. After he had initially presented himself as a nationalist rather than a Shia leader, people blamed him for being too sectarian. The main points of criticism related to his failure to engage with the demands of Sunni protestors. Protests increased even more after Maliki purged the government of Sunnis, whom he accused of having links to terrorism (Sullivan, 2013; Wicken, 2013; Marr and Al-Marashi, 2017). In April 2013, clashes between protestors and security forces resulted in 50 fatalities. This, of course, caused further cleavages.

The government’s failure to address Sunni demands and the violent response to protests paved the way for the emergence of the Islamic State of Iraq and Syria (ISIS). Islamic State of Iraq and Syria had evolved from an insurgent group founded by Abu Musab al-Zarqawi in 2003, which merged with al-Qa’ida in 2004 to form al-Qa’ida in Iraq. Soon the harsh attacks on mainly Muslim victims conducted by ISIS had caused differences between the two groups. The Islamic State in Iraq (ISI) thus established itself as a separate group in 2006. It used terrorism with the goal of establishing a Muslim state and conquering territory. Helped by its anti-Shia stance, it soon attracted Sunni fighters living outside Iraq. It gained more and more power during the civil war in Syria starting from 2011. In 2013, it renamed itself Islamic State of Iraq and Syria (Marr and Al-Marashi, 2017; UNHCR, 2017).

In January 2014, fighting between government forces and tribal fighters broke out in Falluja. Islamic State of Iraq and Syria joined the fight and obtained control of the city. It gained support of local Iraqis by promising to end the corrupt practices of the Iraqi state. Islamic State of Iraq and Syria soon started to invade other cities and seized Mosul in June 2014. Despite the resurging violence, a Shia coalition won the elections in April 2014. This set the stage for the further fragmentation of Iraq. Maliki resigned, leaving his successor, Haidar Abbadi, with many inherited problems. The most drastic challenge was surely the ongoing invasion by ISIS, which carried out massacres all over the country. The Iraqi Security Forces collapsed after ISIS (or IS thereafter) had taken Mosul in June 2014. In the following weeks, ISIS continued to attack areas inhabited by Kurds, Shia, Turkmen, Yazidis, and Christians. It caused wholesale massacres with an estimated death toll of more than 12,000 people (Pettersson and Wallensteen, 2015) and induced thousands of people to flee (Marr and Al-Marashi, 2017). Soon it had conquered almost a third of Iraq’s territory. When ISIS was close to invading Baghdad and attacking sacred Shia sites, thousands of Shia volunteers and some existing Shia militias joined the fight. With the help of US-led airstrikes, starting in August 2014, they finally stopped the advance of ISIS and managed to push it back by mid-2016. Yet, its lingering presence is still a problem (Marr and Al-Marashi, 2017). According to Pettersson and Wallensteen (2015), Iraq was the country with the highest level of violence after Syria in 2014. Although most of the violence targeted Shias, Kurds, Turkmen, Yazidis, and Christians, it was still indiscriminate violence (or “group-selective violence”) in the sense that it was effectively indiscriminate among those in the targeted groups (Straus, 2015; Abdel-Razek and Puttick, 2016; Cetorelli et al., 2017).

The escalating violence in Iraq has induced millions of people to flee from their homes. After the first waves of migration in the 1980s, the flows of migration increased significantly in 1991 and even more so in the mid-2000s. The violence that erupted in 2014, the invasion of ISIS and their taking control of Mosul and large territories in the West caused large streams of forced displacement (World Bank, 2018). It has resulted in the internal displacement of more than 3.3 million people between January 2014 and June 2017, and an estimated 10 million Iraqis require some form of humanitarian aid. Out of the over 230,000 registered refugees in the region, Turkey hosts more than half (UNHCR, 2017).

### Hypotheses

Exposure to violence and, in particular, to such mass-scale and indiscriminate violence as experienced in Syria and Iraq, can trigger strong threat perceptions and anxiety (e.g., Kamans et al., 2011; Schmid and Muldoon, 2013; Hall and Kahn, 2020; Canevello et al., 2021; Hall et al., 2021). For example, observing severe violence or experiencing life-threatening events can shatter people’s basic perceptions about the world as a safe place, one’s role in it, and in particular one’s vulnerability (Janoff-Bulman, 1992; Cassar et al., 2013). Consequently, people may be more sensitive to threats and develop hypervigilance or an obsession with potential danger (Yehuda et al., 2015), or suffer from anxiety, sadness, and post-traumatic stress disorder (PTSD) (Dumke et al., 2021). All these mechanisms are likely to affect people’s behavior and day-to-day decision-making. As a reaction to traumatic experiences, many people withdraw emotionally and from social interaction or respond with reduced social capital (Cassar et al., 2013; Dumke et al., 2021). All these potential reactions to unsettling experiences are likely to reduce trust towards everybody. We thus expect generalized social trust to decrease with conflict exposure.

*H1*: Conflict exposure reduces generalized social trust.

The migration literature suggests that migrants often have high initial trust in the host country’s institutions (Maxwell, 2010; Adman and Strömblad, 2015). Based on this assumption, it would seem reasonable to assume that the refugees trust the Turkish authorities more than Syrian or Iraqi institutions. At the same time, most of the refugees did not have any other choice than to leave their home country. They did not choose Turkey because of their high trust in its institutions, but because fleeing to Turkey was their only way to security. Hence, the assumptions of the migration literature of high initial trust in the host country’s institutions might not hold for our sample. Moreover, many of our subjects experienced violence and conflict, which may have triggered threat perceptions. These threat perceptions and the psychological mechanisms described above are likely to also reduce institutional trust even in the host country. For example, Whitt (2010) finds strong linkages between institutional trust and social trust in post-war Bosnia. Moreover, violence and unrest in Syria and Iraq were largely state-inflicted. This may shape not only trust in institutions in their home countries but affect the refugees’ general perception of state institutions and thus also reduce trust in host country institutions. Furthermore, the political and social situation in Turkey at the time of our survey was not stable. This may exacerbate threat perceptions and trigger negative memories. We thus hypothesize a negative relationship between conflict exposure and trust in state institutions in Turkey.

*H2*: Conflict exposure reduces trust in various institutions in Turkey.

Different forms of conflict exposure may affect trust in different ways (Arnetz et al., 2014; Werner and Skali, 2021). Studies by Hayes and McAllister (2001), Srinivasa Murthy (2007), or Schmid and Muldoon (2013), for example, have shown that the impact of conflict exposure depends on the directness of exposure. Attitudes and behavior may depend on whether one was directly involved or merely has heard about other people’s experiences. Similarly, Macksoud and Aber (1996) find that it makes a difference how actively one was involved. Other studies show that not only the directness of experiences but also their type and severity may trigger different emotions and behavioral reactions. For example, experiences that involve threat to somebody’s life or serious injury may trigger posttraumatic reactions and emotions like fear and distrust. In contrast to this, threats to financial resources or property could trigger anger (Mackie et al., 2000; Cottrell and Neuberg, 2005; Kamans et al., 2011; Lavi et al., 2012). Based on this literature, we hypothesize that more severe experiences have more traumatic consequences and in turn have a stronger impact on trust. More severe could be those experiences that involve emotional pain and active physical involvement. If such experiences trigger stronger threat perceptions or more withdrawal, this should affect social and institutional trust alike. This assumption is also in line with findings by Whitt (2010) that variation in institutional trust is strongly linked to variation in social trust.

*H3a*: More severe forms of conflict exposure have a stronger negative impact on generalized social trust.

*H3b*: More severe forms of conflict exposure have a stronger negative impact on institutional trust.

We hypothesize that high levels of PTSD are correlated with low levels of both social and institutional trust. Typical symptoms of PTSD involve a lack of perceived involvement and empathy (Amaya-Jackson and March, 1995; McKnight et al., 2004; Srinivasa Murthy, 2007). People who have had traumatic experiences may suffer from emotional numbness, in particular in an unstable environment (Werner and Lambsdorff, 2019). This may reduce not only social trust but also the propensity to trust different institutions. At the same time, having undergone a positive psychological change in the form of PTG is also likely to affect trust. As PTG can be manifested in improved and deeper interpersonal relationships (Tedeschi and Calhoun, 2004), we hypothesize that it is likely to go along with increased social trust. Another domain of PTG are a changed philosophy of life and the recognition of new possibilities for one’s life (Tedeschi and Calhoun, 2004), which could correlate with institutional trust.

*H4*: PTSD reduces social trust and institutional trust, while PTG increases both forms of trust.

## Materials and Methods

### Participants

#### Participant Background

We surveyed a community-based sample of 832 refugees from Syria and Iraq in 2016. All these refugees resided in the Central Anatolia region of Turkey, centered around the city of Konya. The city of Konya is often considered the center of Sunni conservatism in Turkey. Social life in Konya is mainly organized along sectarian lines, with local mosques often serving as the centers of communities.

We began our fieldwork in Konya in the neighborhood of Bosna Herceg, an inexpensive area where students and refugees both live. The neighborhood is also in the vicinity of the largest and oldest university in the area, Selcuk University. While the area is very conservative, students tend to be more liberal and not very religious. There are mainly Arabs but also Kurds living in this area. The vast majority are conservative and Sunni but some are Shia as well. As in most parts of Turkey, refugees in this area largely do not live in camps. Most live in small apartments. The typical family is relatively poor and relies substantially on aid. Unemployment is high and working on the black market is common. Discrimination against refugees was unfortunately a common experience, although many reported good relations with their Turkish neighbors.

The majority of the participants in our sample arrived in Turkey between 2010 and 2015. In these years, as described in Section “Brief Summary of the Conflicts in Syria and Iraq”, there had been intense fighting between government and opposition troops in Iraq and Syria, with major cleavages along religious lines (roughly speaking Shia and Alawi vs. Sunni in Syria; Sunni vs. Shia in Iraq; even though ethnic and other group identities doubtlessly played in). Since most Syrian and Iraqi refugees are conservative Sunni Muslims who share the same religion as the majority of Turkey’s population, this is an especially fascinating environment to examine the influence of traumatic events on refugee trust.

#### Participant Recruitment

Running such a study in the field is challenging and requires diligent preparation. We first invested substantial time and effort in building up relationships and gaining trust in the refugee community. Our first approach was to visit NGOs that deliver food and medical aid to refugees in Konya. While refugees were waiting to receive their daily food rations, we approached them to make initial contacts within the community. To get in touch with more educated refugees, we additionally approached people in student cafes. To assist in these efforts, we recruited three Arabic-speaking research assistants who had good connections to the refugee community.

We then put a lot of care into the training of the assistants and the preparation of the research materials. This took place in Konya from November 7–14, 2016. During this time, the assistants became familiar with the research materials. We also asked them for feedback on the survey instrument and, in particular, the Arabic translation. A professional editor of Arabic texts in Sweden had translated the research materials into Arabic. Our research assistants then evaluated the Arabic translations and adapted them to reflect the Arabic dialects spoken in the local refugee community. After that, the professional translator again checked and approved the adapted translations, which were more colloquial. We left all well-established measures of conflict exposure and psychometric scales unchanged, for which we had Arabic translations from the original authors. These well-established measures comprise the Harvard Trauma Questionnaire—Part I, the Posttraumatic Growth Inventory-Short Form (PTGI-SF), and the PTSD-Checklist (PCL-C Short Form). After finalizing the research materials, we recruited eight refugees to test the materials. With these test subjects, the assistants practiced multiple times conducting the survey. The test subjects also provided valuable feedback on the clarity of the instructions and the wording of some questions. We then made minor improvements to address these suggestions. Further information can be found in the Supplementary Material (Section 4).

After the training period, the assistants moved on to different cities depending on their networks. The data collection focused on Central Anatolia, particularly the town of Konya and its Bosna Hersek neighborhood; but also included the towns of Adana, Ankara, Antalya, Balikesir, Hatay, Istanbul, Kahramanmaras, Mersin, and Yalova. The data collection took place between November 15 and December 27, 2016.

When entering a community, the assistants first approached key persons and built up a trusting relationship with them. This was done by explaining the background and purpose of the study with the help of a background information sheet. The key persons then helped the assistants to visit other community members. Whenever we had gained the trust of well-known people, it was much easier to approach other people in the community and ask them to take part in the study. Due to the lack of a sampling frame for refugees living in the area, we used a chain-referral sampling procedure. Upon participation in the study, we asked the participants to put us in contact with other families in their social networks. Almost all people we asked were willing to take part once we had gained the trust of key persons in the community. To further diversify the sample, we additionally approached refugees standing in breadlines, outside aid organizations, in public transportation hubs, at universities, and in front of refugee camps.

We surveyed the participants in the privacy of their own apartments on tablets, using the software Qualtrics. Before starting the survey, participants gave their informed consent to participating in the study. Participants were informed that they could break off participation at any time. They also learned that the study was completely anonymous and that we would not store any identifying information. The project was approved by the Uppsala Ethical Review Board (Etikprövningsnämden, EPN) in May 2016 (Dnr 2016/189). In accordance with our ethical approval, we further excluded 30 participants who stated that their age was under 18. We ended up with a sample of 802 subjects.

#### Demographic Characteristics

Table 1 provides an overview of this sample. 40.7 percent of our participants are female, 40.9% come from Syria, the remaining 59.1% from Iraq. The vast majority (82.1%) describe the character of their location of origin as urban rather than rural. The majority of our participants is between 18 and 34 years old, has a comparably high level of education, and arrived in Turkey between 2010 and 2015. The sample is quite diverse when it comes to their perceived social and economic status in their country of origin. Their mean self-reported socioeconomic status in their country of origin is at 4.5, which is slightly below the midpoint of the scale from 0 to 10. The sample is also quite heterogeneous when it comes to the question of whether they believe they will ever move back to their country of origin to live there permanently. 28.3% believe they will never move back. Out of the remaining 71.7% who believe that they will move back at some point, the largest group believes they will move back 5–10 years from now. As our assistants were college students, the sample is somewhat skewed toward younger and more educated people.

**Table 1:** Sample overview.

**Demographics**		**%**		**Perceived prospects of moving back to country of origin**		**%**	
Female		40.7		No, I do not think I will move back		28.34	
from Syria		40.9		Yes, 0–4 years from now		26.30	
from Iraq		59.1		Yes, 5–10 years from now		33.67	
from urban region		82.1		Yes, when I am old		11.69	
			
**Age**	**%**	**Education**	**%**	**Arrived in Turkey**	**%**	**Social and economic status in country of origin**	**%**
18–24	35.71	No formal education	3.13	1969 or earlier	0.26	Worst off	3.15
25–34	29.21	<6 years of schooling	3.75	1986–1989	0.39	.	11.22
35–44	15.31	6 years of schooling	6.75	2000–2005	0.78	.	13.37
45–54	9.69	9 years of schooling	13.13	2006–2009	3.23	.	10.97
55–64	5.87	12 years of schooling	21.63	2010–2015	82.56	.	12.86
65–74	2.93	>12 years of schooling	51.63	2016-today	12.79	.	15.38
75–84	0.77					.	8.20
>85	0.51					.	8.32
						.	9.08
						.	4.16
						Best off	3.28

### Measures

#### Dependent Variables

##### Generalized Social Trust

As a measure of generalized social trust, we use the World Values Survey question: “Generally speaking, would you say that most people can be trusted, or that you cannot be too careful in dealing with people?.” This way of asking the question is standard in studies that rely upon the World Values Survey and General Social Survey, and has been deployed in over 500 research papers according to Sapienza et al. (2013). Possible answers included “Most people can be trusted,” “It depends,” “Cannot be too careful,” “Most people cannot be trusted” and “I do not know.” This approach carefully disaggregates caution (cannot be too careful) and trust preferences, which are commonly conflated in measures of social trust (Soroka et al., 2003; OECD, 2017). These answer categories are frequently used in the literature—most recently by Moore and Carpiano (2020).^3^ Following previous literature, we coded generalized social trust as a dummy variable that takes on the value 1 if a subject answered “Most people can be trusted” and 0 otherwise (if the answer was “Cannot be too careful”; “it depends”; “most people cannot be trusted” or “I do not know). This approach allows us to compare those who indicated high trust by selecting “most people can be trusted” to those who indicated low trust by selecting any of the other four categories (e.g., Bassett and Moore, 2013a, 2013b; see also Lindström, 2009; Legh-Jones and Moore, 2012; Moore et al., 2014, 2016; Child et al., 2017; Wu et al., 2018). In Supplementary Table 3, we provide robustness checks with different ways of coding this trust question (both as dichotomous and ordinal variable) and show that our results are robust to various approaches to coding responses.

##### Institutional Trust

Drawing upon previous measures of institutional trust (see, e.g., Newton et al., 2018 for a review), we constructed a survey item that captures subjects’ trust toward a comprehensive set of state institutions. Subjects were asked: “How much do you personally trust and feel confidence in each of the following institutions in Turkey?,” with possible answers ranging from “do not trust at all” to “completely trust” on a 5-point Likert-scale. The set of state institutions included: The courts, the police, politicians, political parties, the parliament, and the national government. Distributions of answers for all items are provided in Table 2.

**Table 2:** Distributions and summary statistics for key dependent and independent variables.

**Generalized social trust**					**%**	
I do not know					5.06	
Most people cannot be trusted					19.47	
It depends					35.15	
Cannot be too careful					11.76	
Most people can be trusted					28.57	
*N*					791	
	
**Institutional trust**	**Courts %**	**Police %**	**Politicians %**	**Parties %**	**Parliament %**	**Government %**
Do not trust	2.19	2.73	10.82	12.21	3.89	3.65
Do not trust very much	5.29	7.79	19.30	24.68	16.34	10.94
I do not know	12.77	8.57	18.51	19.87	19.58	17.58
Trust somewhat	31.23	31.43	24.12	20.39	25.68	24.87
Trust completely	48.52	49.48	27.25	22.86	34.50	42.97
*N*	775	770	767	770	771	768
	
**Exposure and consequences**	**N**	**Mean**	**Std. Dev.**		**Min**	**Max**
War exposure (HTQ)	802	4.70	3.98		0	16
PTS (PCL-C)	762	17.27	5.88		5	30
PTG (PTGI-SF)	759	41.58	8.65		5	60

#### Independent Variables

##### War Exposure

**Figure 1:**
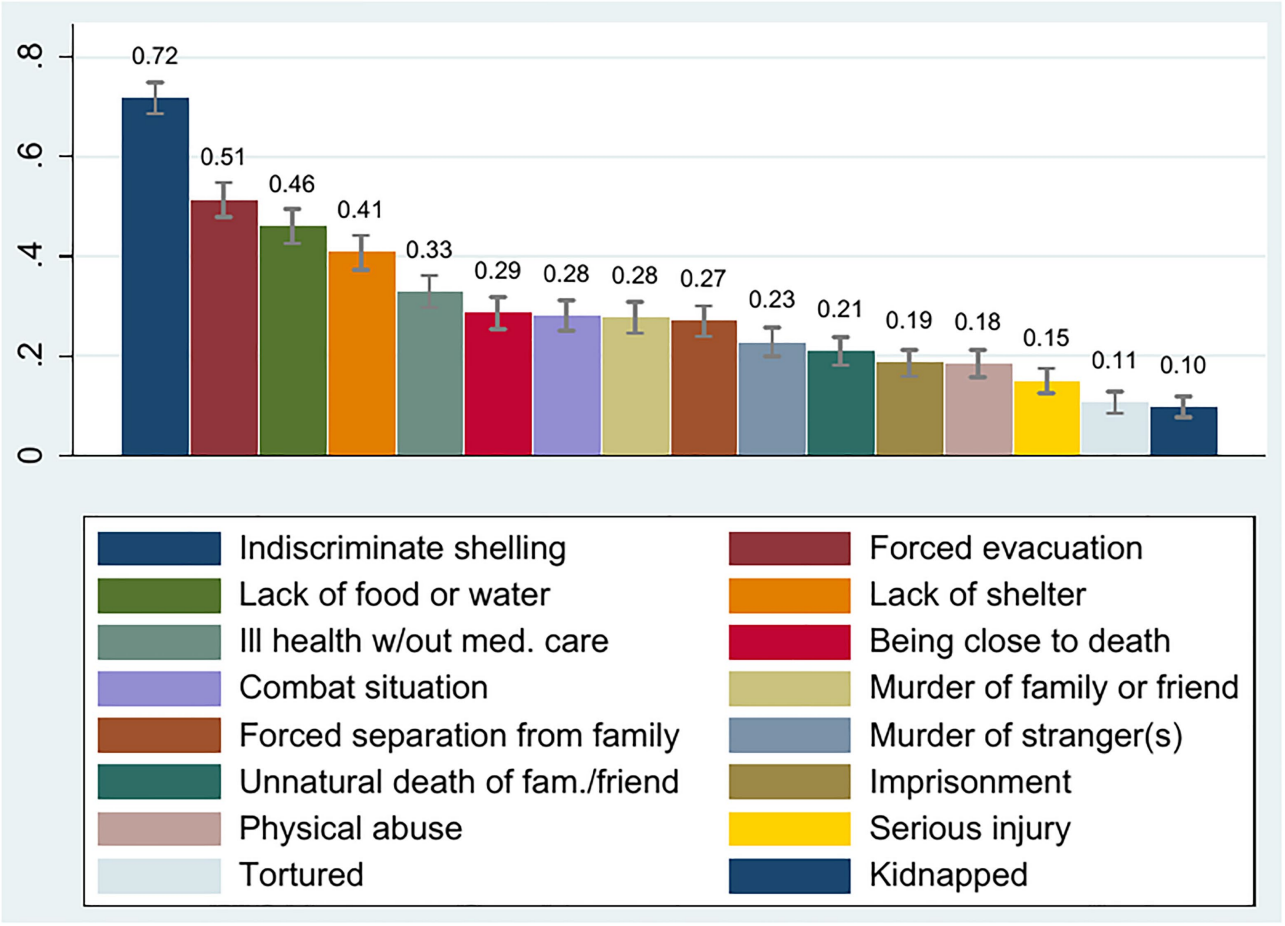
Sixteen items of the Harvard Trauma Questionnaire. Capped ranges indicate 95% confidence intervals.

To measure exposure to violence, we used a version of the Harvard Trauma Questionnaire with an event list of 16 items deemed to be relevant to Syrian and Iraqi refugees. Subjects were asked: “Please indicate whether you have experienced any of the following in your life before arriving in Turkey. Select all that apply.”^4^ This is standard practice for measuring conflict exposure (Netland, 2005; Schmid and Muldoon, 2013). Figure 1 displays the 16 events and the percentage of subjects who had experienced the respective event. It is clearly visible that the vast majority of our subjects (72%) has experienced indiscriminate shelling. This emphasizes the as-if randomness of conflict exposure and supports our analysis. More than half of our participants were forcibly evacuated, which also underlines that migration was not voluntary. Many subjects (more than one third) have also experienced a lack of food or water, a lack of shelter, or ill health without medical care. The picture looks very similar when we display it separately for Syrian and Iraqi refugees. From the 16 events of the Harvard Trauma Scale, we formed a cumulative index, which sums up all the events a subject has experienced (see, for example, Bellows and Miguel, 2009, or Dubow et al., 2010 for a justification of an additive index). We provide summary statistics of this index in Table 2. A histogram can be found in Supplementary Figure 1.

##### Posttraumatic Stress

Symptoms of Posttraumatic Stress were measured with the civilian version of the PTSD Checklist (PCL-C). This checklist asks subjects: “Below is a list of problems and complaints that people sometimes have in response to stressful life experiences. Please indicate how much you have been bothered by each problem in the last month” with possible answers “Not at all,” “A little bit,” “Moderately,” “Quite a bit” and “Extremely.” The six problems comprised: “Repeated disturbing memories, thoughts, or images of a stressful experience from the past,” “Feeling very upset when something reminded you of a stressful experience from the past,” “Avoid activities or situations because they remind you of a stressful experience from the past,” “Feeling distant or cut off from other people,” “Feeling irritable or having angry outbursts,” “Having difficulty concentrating.” We formed an additive index of these symptoms. Summary statistics can be found in Table 2 and a histogram in Supplementary Figure 2. In this index, a score of 14 or more is considered suggestive of post-traumatic stress *disorder* (Lang and Stein, 2005). 70.9% of our sample fulfill this criterion.

##### Posttraumatic Growth

Similarly, we use an additive index of the 10 items of the Posttraumatic Growth Inventory-Short Form (PTGI-SF, Cann et al., 2010), which asks: “For each of the statements below, please indicate the degree to which this change occurred in your life as a result of all that has happened.” The 10 items were “I changed my priorities about what is important in life,” “I have a greater appreciation for the value of my own life,” “I am able to do better things with my life,” “I have a better understanding of spiritual matter,” “I have a greater sense of closeness with others,” “I have established a new path for my life,” “I know better that I can handle difficulties,” “I have a stronger religious faith,” “I discovered that I’m stronger than I thought I was,” “I learned a great deal about how wonderful people are.” Possible answers comprised “Not at all,” “To a very small degree,” To a small degree,” “To a moderate degree,” “To a great degree” and “To a very great degree.” We provide summary statistics of the additive index in Table 2, and a histogram in Supplementary Figure 3. Sixty-five percent of our sample can be classified as having moderate to high levels of posttraumatic growth (cutoff values taken from Jansen et al., 2011).

## Results

### Exposure to Violence and Social Trust

We first investigate the relationship between exposure to violence and generalized social trust.

**Figure 2:**
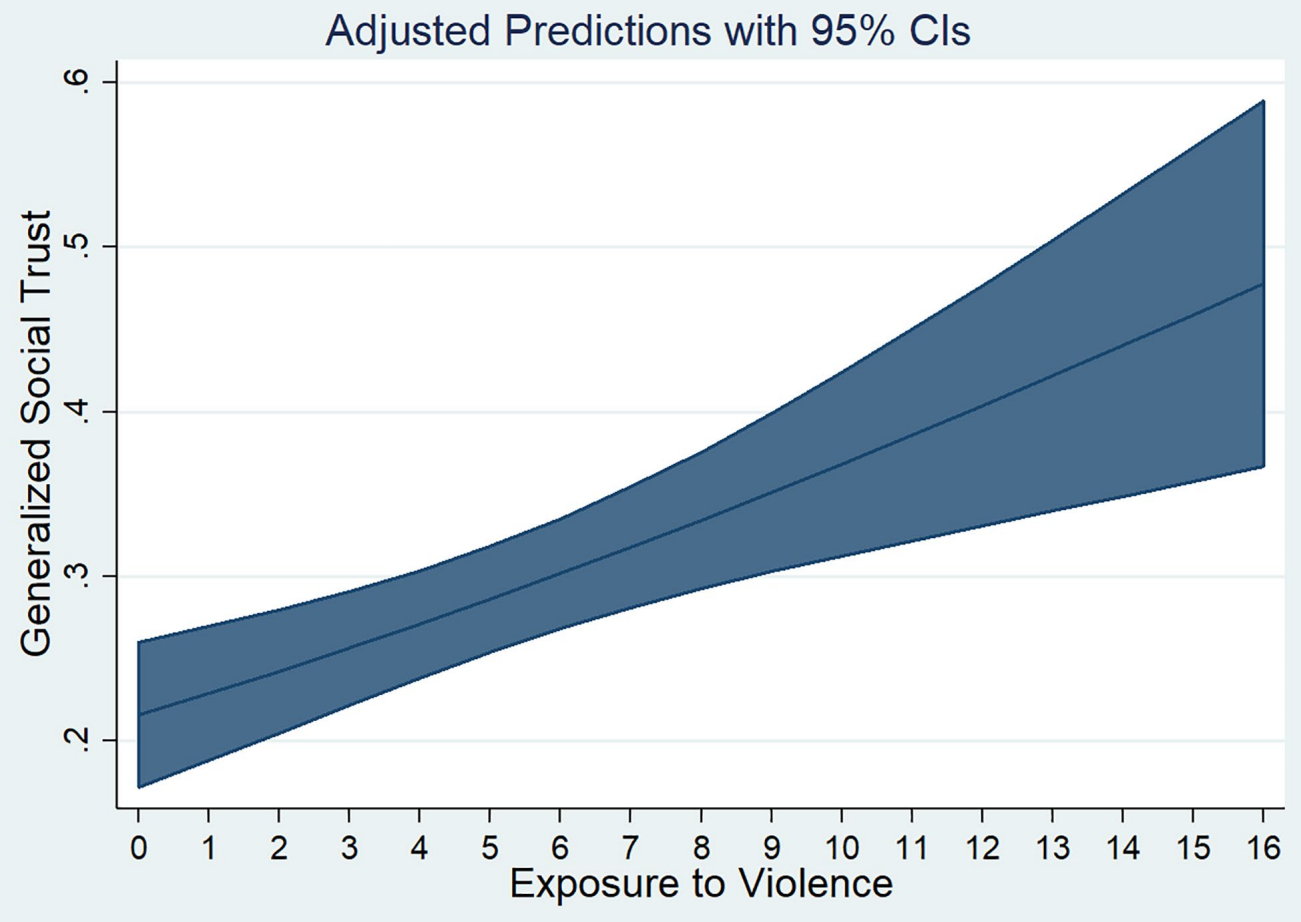
Relationship between exposure to violence and generalized social trust.

Table 3 reports the results of logistic regressions with our binary measure of generalized social trust as the dependent variable and exposure to violence as our main independent variable of interest. In all models, we report marginal effects at mean values of the regressors. In the first column, we simply regress generalized social trust on exposure to violence, without any further controls. It indicates a significant positive relationship between exposure to violence and generalized social trust: Having experienced one additional event of the 16 items in the Harvard Trauma Scale increases the probability of believing that people generally can be trusted by ~1.5%.^5^ Figure 2 plots the predictions and 95% intervals of this bivariate regression. It illustrates the positive correlation between exposure to violence and the probability of believing that most people can be trusted. This seems counterintuitive at first sight, but is consistent with the literature on posttraumatic growth (e.g., Tedeschi and Calhoun, 1996, 2004) and with empirical findings of increased social capital after conflict (Lyons et al., 1998; Bellows and Miguel, 2009; Blattman, 2009; Voors et al., 2012; Gilligan et al., 2014; De Luca and Verpoorten, 2015a; Bauer et al., 2016). During the conflict, subjects may have had experiences of mutual support and solidarity that increased their social trust. The finding could also be supported by the fact that subjects had managed to flee their country and were in a safe place when they answered the trust question.^6^

**Table 3:** Exposure to violence and generalized social trust.

Variables	(1)	(2)	(3)
	Dep. variable: Social Trust Marginal Effects		
Exposure to violence	0.02^***^	0.02^***^	0.02^***^
	(0.004)	(0.004)	(0.005)
Female		−0.01	−0.03
		(0.03)	(0.04)
Age		0.007	0.009
		(0.01)	(0.01)
Syria		−0.1^***^	−0.1^***^
		(0.04)	(0.04)
Further controls			✓
Observations	791	765	726
Pseudo *R*^2^	0.016	0.031	0.054

Logistic regressions, Marginal effects at mean values, Robust standard errors in parentheses. Dependent variable: Social Trust (binary measure, 0 = “most cannot be trusted”/“cannot be too careful”/“it depends”/“I do not know”; 1 = “most people can be trusted”). Independent variables: Exposure: Additive index of the 16-item Harvard Trauma Questionnaire. Female: dummy variable indicating respondent’s gender; 0 = male, 1 = female. Age: proxy of age; 1 = 18–24, 2 = 25–34, 3 = 35–44, 4 = 45–54, 5 = 55–64, 6 = 65–74, 7 = 75–84, 8 = 85 or older. Syria: dummy variable indicating respondent’s country of origin; 0 = Iraq, 1 = Syria. Further controls: Social/economic status: Self-reported status in country of origin, 0 (the worst off) … 10 (the best off). Education: Indicator variable, 1 = no formal education, 2 = < 6 years of schooling, 3 = 6 years of schooling, 4 = 9 years of schooling, 5 = 12 years of schooling, 6 = > 12 years of schooling. Urban: Dummy variable indicating whether a respondent self-reported to come from a rural (0) or urban (1) region. Arrived in 2016: Dummy variable indicating whether a respondent arrived in Turkey in 2016 (1) or not (0) region. Return: Indicator variable on belief to move back; 0 = No, I do not think I will move back; 1 = Yes, 0–4 years from now; 2 = Yes, 5–10 years from now; 3 = Yes, when I am old. ^*^p < 0.10; ^**^p < 0.05.

*** *p* < 0.01.

In columns (2) and (3) of Table 3, we show that the effect of exposure to violence on generalized social trust is robust to the inclusion of various control variables.^7^ In fact, the effect gets even larger when the controls are considered. We can therefore clearly reject hypothesis 1 and find the opposite.

**Result 1:** Conflict exposure increases generalized social trust among refugees.

### Exposure to Violence and Trust in Turkish Institutions

Second, we investigate the impact of exposure to violence on different kinds of institutional trust.

**Figure 3:**
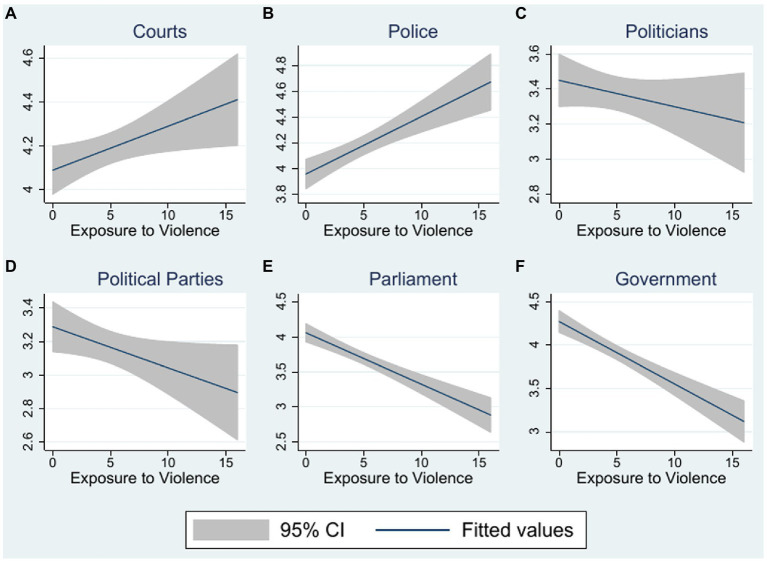
Relationship between exposure to violence and institutional trust.

Table 4 presents OLS^8^ regressions with the different measures of institutional trust as the dependent variable. The relationship between exposure to violence and trust in (A) courts, (B) police, (C) politicians, (D) political parties, (E) parliament and (F) the government in Turkey is illustrated in Figure 3 by plotting the predictions and 95% intervals of the bivariate regressions from Table 4. The first notable finding from Table 4 and Figure 3 is that exposure to violence has diverse effects on trust in different kinds of institutions: Exposure to violence has a significantly positive effect on trust in courts (1), and trust in the police (2). At the same time, exposure to violence has significantly negative effects on trust in (4) political parties, the parliament (5) and in the government (6). Table 5 presents OLS regressions of the measures of institutional trust on conflict exposure with the same battery of additional controls as used in Table 3.

**Table 4:** Exposure to violence and institutional trust in the settlement country.

	(1)	(2)	(3)	(4)	(5)	(6)
	Courts	Police	Politicians	Parties	Parliament	Government
Exposure to violence	0.02^**^	0.04^***^	−0.02	−0.02^**^	−0.07^***^	−0.07^***^
	(0.008)	(0.009)	(0.01)	(0.01)	(0.01)	(0.01)
Constant	4.1^***^	4.0^***^	3.4^***^	3.3^***^	4.1^***^	4.3^***^
	(0.06)	(0.06)	(0.08)	(0.08)	(0.07)	(0.06)
Observations	775	770	767	770	771	768
*R* ^2^	0.007	0.029	0.002	0.005	0.059	0.061

OLS regressions, robust standard errors in parentheses. Dependent variables: Self-reported trust in institutions in Turkey, 1 = “Do not trust at all,” 2 = “Do not trust very much,” 3 = “I do not know,” 4 = “Trust somewhat,” 5 = “Trust completely.” Independent variable: Exposure: Additive index of the 16-item Harvard Trauma Questionnaire. ^*^p < 0.10;

** *p* < 0.05;

*** *p* < 0.01.

**Table 5:** Exposure to violence and institutional trust in the settlement country with controls.

	(1)	(2)	(3)	(4)	(5)	(6)
	Courts	Police	Politicians	Parties	Parliament	Government
Exposure to violence	0.03^***^	0.04^***^	−0.02	−0.02	−0.07^***^	−0.07^***^
	(0.010)	(0.01)	(0.01)	(0.01)	(0.01)	(0.01)
Female	−0.1^*^	−0.05	0.006	0.06	−0.3^***^	−0.2^***^
	(0.08)	(0.08)	(0.10)	(0.10)	(0.09)	(0.08)
Age	0.03	−0.01	−0.01	−0.07^*^	0.04	0.04
	(0.03)	(0.03)	(0.04)	(0.04)	(0.03)	(0.03)
Syria	−0.3^***^	−0.09	−0.6^***^	−0.5^***^	−0.2^*^	−0.2^**^
	(0.09)	(0.10)	(0.1)	(0.1)	(0.1)	(0.1)
Further controls	✓	✓	✓	✓	✓	✓
Constant	4.2^***^	3.6^***^	3.0^***^	3.1^***^	4.0^***^	3.7^***^
	(0.2)	(0.3)	(0.3)	(0.3)	(0.3)	(0.4)
Observations	713	710	709	711	712	710
*R* ^2^	0.097	0.090	0.171	0.204	0.182	0.208

OLS regressions, robust standard errors in parentheses. Dependent variables: Self-reported trust in institutions in Turkey, 1 = “Do not trust at all,” 2 = “Do not trust very much,” 3 = “I do not know,” 4 = “Trust somewhat,” 5 = “Trust completely.” Independent variables: Exposure: Additive index of the 16-item Harvard Trauma Questionnaire. Female: dummy variable indicating respondent’s gender; 0 = male, 1 = female. Age: proxy of age; 1 = 18–24, 2 = 25–34, 3 = 35–44, 4 = 45–54, 5 = 55–64, 6 = 65–74, 7 = 75–84, 8 = 85 or older. Syria: dummy variable indicating respondent’s country of origin; 0 = Iraq, 1 = Syria. Further controls: Social/economic status: Self-reported status in country of origin, 0 (the worst off) … 10 (the best off). Education: Indicator variable, 1 = no formal education, 2 = <6 years of schooling, 3 = 6 years of schooling, 4 = 9 years of schooling, 5 = 12 years of schooling, 6 => 12 years of schooling. Urban: Dummy variable indicating whether a respondent self-reported to come from a rural (0) or urban (1) region. Arrived in 2016: Dummy variable indicating whether a respondent arrived in Turkey in 2016 (1) or not (0) region. Return: Indicator variable on belief to move back; 0 = No, I do not think I will move back; 1 = Yes, 0–4 years from now; 2 = Yes, 5–10 years from now; 3 = Yes, when I am old. The complete table with all coefficients can be found in Supplementary Table 9.

* *p* < 0.10;

** *p* < 0.05;

*** *p* < 0.01.

The effect on trust in political institutions (political parties, parliament and government) in Turkey is negative. The negative effect is particularly strong for the government.^9^ This may be related to the fact that the refugees were fleeing misgovernment and oppressive regimes. Syrian civilians were mainly terrorized by the Assad regime and its supporters. As a consequence, they might distrust the parliament and the government in their home country, which may translate into distrust in such institutions in Turkey. In Iraq, Assad’s and Maliki’s misgovernment as well as Maliki’s violent reaction to civilian protests had paved the way for the emergence of ISIS. The refugees might thus also be more alerted by the tensions in Turkey in 2016 and react with distrust toward the Turkish government. Trust in courts and in particular in the police, on the other hand, increases with exposure to violence. Thus, the effects of conflict exposure on trust in the police and courts go in the same direction as its effects on social trust. We can interpret this as support for the argument that trust in order-keeping and policy-implementing institutions is important for generalized social trust (Rothstein and Stolle, 2008). It also lends support to the assumption that institutions such as the police or courts are often expected to be relatively impartial (Rothstein and Stolle, 2008; Dinesen and Bekkers, 2017).

In Supplementary Tables 17, 18 we show that the positive effects on social trust are similar for refugees from Syria and Iraq, but that the negative effects for political trust are strongly driven by refugees from Iraq.^10^ At the same time, the positive effects for courts and the police are more pronounced for refugees from Syria. Since the rebels established their own administrative councils and courts and formed civilian police forces to protect the people, Syrian refugees may have felt the importance of enhanced security and police protection.

We can thus reject Hypothesis 2 with respect to courts and the police, but not with respect to all political institutions.

**Result 2:** Conflict exposure decreases trust in Turkish political parties, parliament and government and increases trust in Turkish courts and the police.

### Different Subtypes of Conflict Exposure

**Figure 4:**
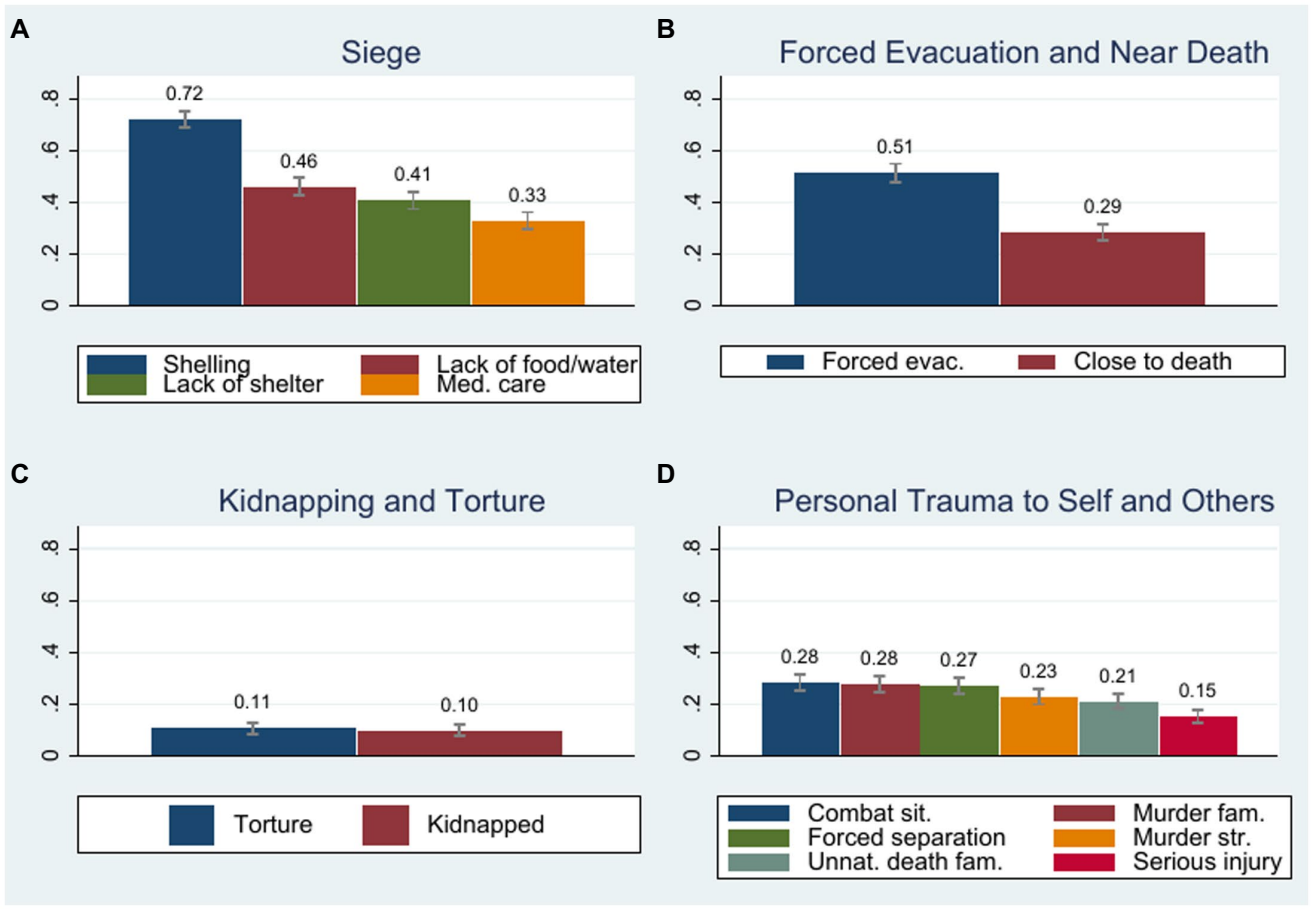
Four subtypes of traumatic events. Capped ranges indicate 95% confidence intervals.

Literature on exposure to violence usually examines the aggregate impact of different experiences. In the following, we go beyond this by investigating whether different types of conflict experiences have different effects on trust. This helps us to get a more refined picture of potentially heterogeneous effects of different exposure types and to illuminate potential mechanisms. Arnetz et al. (2014), for example, show that trauma subtypes are better predictors of refugees’ mental health outcomes than a cumulative trauma index. This might also hold for predicting trust. To investigate hypothesis 3, we thus use principal component analysis to derive four factors. Due to dual loading on multiple factors, we discard the items “imprisonment” and “physical abuse.” We follow the procedures described by Arnetz et al. (2014) and use oblique rotation. This is because the four factors are likely to correlate moderately with one another and in order not to force the factors to be independent. We include all items with a primary loading >0.30 on the respective factor. We name the four factors based on their content. Factor (1) – Siege–includes Lack of food or water, Ill health without medical care, Lack of shelter, Indiscriminate shelling or bombing. Factor (2) – Forced Evacuation and Close to Death–comprises Forced evacuation and Being close to death. Factor (3) – Kidnapping and Torture–consists of having been kidnapped or tortured. Factor (4) – Personal Trauma to Self and Others–comprises Serious injury, Combat situation, Forced separation from family, Murder of family member or friend, Unnatural death of family member or friend and Murder of stranger or strangers. The PCA outputs are provided in Supplementary Table 2. Figure 4 illustrates the four subtypes, the items they comprise, and the percentage of our sample that has experienced each respective item. Following Kling et al. (2007), we calculate each factor by taking the sum of the z-scores of each item divided by the standard deviation of this sum, such that each factor has a mean of 0 and a standard deviation of 1.

Following similar classifications by Macksoud and Aber (1996), we regard “Personal Trauma to Self and Others” as well as “Kidnapping and torture” as the most severe and personal factors. These clusters of experiences also imply a very active involvement. “Kidnapping and torture” is physically very direct. “Personal trauma to self and others” involves very personal emotional pain. Both forms involve aggression and damage inflicted on individuals. As entire regions in Syria and Iraq were forcefully evacuated, forced evacuation and the perception of having been close to death are less directed toward an individual and may thus not have as strong an impact on trust. Furthermore, our subjects with these experiences have escaped death or serious injury. We thus regard “Forced evacuation and close to death” as less personal than “Personal trauma to self and others” or “Kidnapping and torture.” The items summarized in the cluster “Siege” comprise hardship and deprivation that happened to many people, even in areas that did not experience combat situations. Hence, we interpret this as the least direct and personal experience.

Table 6 shows the results of regressions that include the four subtypes of traumatic events to explain trust (logistic regressions for the binary measure of generalized social trust and OLS regressions for the measures of institutional trust). “Personal trauma to self or others” is strongly associated with higher social trust. It turns out that these very personal experiences are the driver of the positive impact of conflict exposure on social trust we found in Table 3. Apart from “personal trauma to self or others,” there is no other significant predictor of social trust. At the same time, “personal trauma to self or others” erodes trust in political institutions. It is negatively related to trust in politicians, parties, the parliament, and the government. “Personal trauma to self or others” also appears to be the driver of the negative impact of conflict exposure on political trust. “Kidnapping or torture” is negatively related to trust in the parliament. This could be explained by the fact that state actors were responsible for a lot of human rights abuse in the refugees’ countries of origin (Human Rights Watch, 2018) and these experiences translate into distrust towards Turkish state actors. At the same time, the share of subjects who had experienced kidnapping or torture is relatively small and this result should thus be treated with caution. Interestingly, both “siege” and “forced evacuation and near death” are associated with higher trust in all the institutions. These less personal experiences not only have no detrimental effect on institutional trust but even a positive one (which is, however, much smaller in size than the negative effects).

**Table 6:** The impact of subtypes of traumatic events on trust.

	(1)	(2)	(3)	(4)	(5)	(6)	(7)
	Social Trust	Courts	Police	Politicians	Parties	Parliament	Government
Personal trauma self/others	0.12^***^	−0.019	−0.042	−0.28^***^	−0.42^***^	−0.56^***^	−0.51^***^
	(0.02)	(0.05)	(0.05)	(0.06)	(0.06)	(0.05)	(0.05)
Kidnapping & torture	0.0013	−0.021	0.010	−0.025	0.012	−0.088^**^	−0.034
	(0.02)	(0.04)	(0.04)	(0.05)	(0.05)	(0.04)	(0.05)
Forced evacuation, near death	−0.027	0.099^**^	0.094^**^	0.096^*^	0.16^***^	0.16^***^	0.16^***^
	(0.02)	(0.04)	(0.04)	(0.05)	(0.05)	(0.05)	(0.04)
Siege	−0.0077	0.089^**^	0.17^***^	0.17^***^	0.24^***^	0.19^***^	0.10^**^
	(0.02)	(0.04)	(0.04)	(0.06)	(0.05)	(0.04)	(0.04)
Constant		4.33^***^	3.79^***^	2.91^***^	3.03^***^	3.66^***^	3.36^***^
		(0.2)	(0.3)	(0.3)	(0.3)	(0.3)	(0.4)
Socio-demographic controls	✓	✓	✓	✓	✓	✓	✓
Observations	726	713	710	709	711	712	710
*(Pseudo) R* ^2^	0.071	0.106	0.105	0.197	0.259	0.276	0.276

Robust standard errors in parentheses. (1) Logistic regression, marginal effects at means; (2)–(7): OLS. Dependent variables: (1) Social Trust (binary measure, 0 = “most cannot be trusted”/“cannot be too careful”/“it depends”/“I do not know”; 1 = “most people can be trusted”). (2)–(7) Self-reported trust in institutions in Turkey, 1 = “Do not trust at all,” 2 = “Do not trust very much,” 3 = “I do not know,” 4 = “Trust somewhat,” 5 = “Trust completely.” Independent variables: Four types of conflict experiences derived by PCA; sum of z-scores divided by s.d. of the sum. Personal Trauma to Self and Others: Includes having experienced Serious injury, Combat situation, Forced separation from family, Murder of family member or friend, Unnatural death of family member or friend and Murder of stranger or strangers. Kidnapping and Torture: having been kidnapped or tortured. Forced Evacuation and Close to Death: Having been forcibly evacuated or close to death. Siege: includes Lack of food or water, Ill health without medical care, Lack of shelter, Indiscriminate shelling or bombing. Controls: Gender, age, Syria, social/economic status, education, coming from urban/rural area, year of arrival, prospects of moving back. The complete table with all coefficients can be found in Supplementary Table 14.

* *p* < 0.10;

** *p* < 0.05;

*** *p* < 0.01.

We thus can partly reject hypothesis 3a. While more severe or personal experiences do explain our findings on social trust, the effect is positive and not negative as we had expected. We can only partly reject hypothesis 3b: More severe forms of conflict exposure have a stronger negative impact on trust in political institutions. Taken together, we can conclude the following:

**Result 3:** More severe or personal experiences of violence explain Results 1 and 2.

Is it a traumatic reaction that drives our findings? Table 7 displays regressions to investigate the impact of symptoms of Post-Traumatic Stress Disorder on trust (logistic regressions for the binary measure of generalized social trust and OLS regressions for the measures of institutional trust). If subjects suffer from PTSD, this could negatively affect their propensity to trust. We do not find a significant correlation between PTSD and social trust. A typical reaction to trauma is emotional withdrawal, which may explain why social trust is not positively affected. We do find a significant negative correlation between PTSD and trust in the police, courts, politicians, parties, the parliament and the government – even though some of the coefficients are small in size and the result for police is only marginally significant. Hence, PTSD may be the mechanism that at least partly explains reduced institutional trust in response to war exposure. Refugees suffering from PTSD may be particularly sensitive to the political instability in Turkey and the potential for repression.

**Table 7:** The impact of symptoms of posttraumatic stress on trust.

	(1)	(2)	(3)	(4)	(5)	(6)	(7)
	Social Trust	Courts	Police	Politicians	Parties	Parliament	Government
PTSD	0.0022	−0.025^***^	−0.013^*^	−0.026^***^	−0.019^**^	−0.054^***^	−0.064^***^
	(0.003)	(0.007)	(0.007)	(0.009)	(0.009)	(0.008)	(0.007)
Constant		4.65^***^	3.93^***^	3.37^***^	3.38^***^	4.69^***^	4.55^***^
		(0.3)	(0.4)	(0.4)	(0.4)	(0.4)	(0.4)
Controls	✓	✓	✓	✓	✓	✓	✓
Observations	700	691	689	689	691	690	690
*(Pseudo)R* ^2^	0.030	0.110	0.076	0.195	0.215	0.194	0.246

Robust standard errors in parentheses. (1) Logistic regression, marginal effects at means; (2)–(6): OLS. Dependent variables: (1) Social Trust (binary measure, 0 = “most cannot be trusted”/“cannot be too careful”/“it depends”/“I do not know”; 1 = “most people can be trusted”). (2)–(6) Self-reported trust in institutions in Turkey, 1 = “Do not trust at all,” 2 = “Do not trust very much,” 3 = “I do not know,” 4 = “Trust somewhat,” 5 = “Trust completely.” Independent variable: Additive index of 6 symptoms of posttraumatic stress according to PCL-C, for each 1 = “Not at all,” 2 = “A little bit,” 3 = “Moderately,” 4 = “Quite a bit,” 5 = “Extremely.” Controls: Gender, age, Syria, social/economic status, education, coming from urban/rural area, year of arrival, prospects of moving back. The complete table with all coefficients can be found in Supplementary Table 15.

* *p* < 0.10;

** *p* < 0.05;

*** *p* < 0.01.

Additionally, we investigate the effects of posttraumatic growth. While we do find evidence that about 65% of our subjects have experienced changes in their lives that can be described as moderate to high PTG, this explains neither the increased social trust nor the increased trust in courts or the police resulting from experiences of severe war exposure.^11^ This non-finding provides support for alternative explanations that focus on the role of positive experiences. People who have experienced personal trauma to self and others are also most likely to have experienced solidarity. For example, they may have been consoled by others after the murder or unnatural death of a family member or friend, or have received care after their serious injury. They may have found other people who supported them after the forced separation from their family. Such positive experiences may have helped them to keep their trust in other people, despite their negative experiences. While we cannot rule out that a phenomenon like posttraumatic growth is a possible explanation for our findings, at least the clinical measure of posttraumatic growth does not explain the increased social trust and trust in courts and the police. We thus consider experiential explanations to be more likely.

We can hence reject Hypothesis 4 with respect to PTG. Concerning PTSD, it can be rejected for social trust, but cannot be rejected for most types of institutional trust. We find confirmatory evidence that PTSD is negatively related to trust in institutions, particularly political institutions.

**Result 4:** PTG does not increase generalized social trust nor political trust. Post-traumatic stress is not related to generalized social trust, but to decreased institutional trust.

### Robustness Checks

Potential concerns might relate to different forms of self-selection that could have impacted our results. First, could self-selection into migration or different reasons for migration distort our results? For example, could our subjects have high trust in Turkish institutions because they chose Turkey as their destination country? Second, could our results be driven by a potential “survival fallacy” in the sense that people who managed to escape from war differ from people who did not? We believe such concerns to be unfounded for several reasons. First of all, our subjects are forcibly displaced and not voluntary migrants. Hence, their motives for migration should not play a major role, because migration was by and large not voluntary. Moreover, we do not intend to examine different cultural backgrounds or differences in the decision to migrate. Instead, we look at individual differences in conflict exposure within one sample of subjects who have all been forcibly displaced and are now in the same setting in the same country. Similar arguments hold for the survival fallacy. It might well be the case that the positive relationship we find between conflict exposure and various forms of trust is driven by positive experiences people had during the war, and that these are specific to those who managed to flee. Yet, this is not a concern for us. Our intention is not to compare people who managed to flee to those who had to stay behind, but we are interested in variations in conflict exposure among refugees who managed to reach Turkey.

Third, could subjects with certain characteristics have self-selected into conflict exposure and at the same time be more trusting of other people or certain institutions? This is an obvious econometric concern facing all studies on conflict exposure. Even though we cannot randomly vary the level of conflict, violence in both countries was widespread and indiscriminate. This makes people less likely to have self-selected into exposure to conflict. We can hence deem conflict exposure as-if-random. Nevertheless, we take some analytical steps to account for potential endogeneity of conflict exposure (following procedures described, for example, by Bellows and Miguel (2009) or Bauer et al. (2016)). First, we show that our main findings are statistically robust and do not change when we control for various potentially relevant subject and household characteristics (including prewar characteristics such as socio-economic status in the home country, or whether subjects lived in urban or rural areas that might have been targeted differently). The controls also include measures of personality traits and political orientation as well as interactions of conflict exposure with employment status (details can be found in the Supplementary Material, Supplementary Tables 4, 7 for Generalized Social Trust, and Supplementary Tables 8–10 for Institutional Trust). The robustness of our results makes it unlikely that some omitted variable that has a confounding effect is driving our results. Second, we run regressions for subsamples that are less likely to have been purposely targeted or self-selected into violence (see Supplementary Table 6a for Generalized Social Trust and Supplementary Tables 11, 12 for Institutional Trust): We run regressions only for those subjects who indicated having experienced indiscriminate shelling or bombing or who reported forced evacuation. We also follow the standard approach of using subsamples of young people, in which victimization is arguably more random than in the full sample due to their age. Third, we run separate regressions for further subsamples and compare, among others, majority groups to minority groups. With this procedure, we make sure that our results are not simply driven by a specific subsample. For example, we run separate regressions for the largest religious group, Sunni Muslims, and all other religious groups, as well as for the largest ethnic group, Arabs, and all other ethnic groups. With the help of Wald-tests for the equality of coefficients, we show that the impact of conflict exposure is not significantly different for different sub-samples (Supplementary Tables 6b,c). Taken together, our identification strategy and analysis mitigate concerns of endogeneity and make a convincing case that we have identified a real causal impact.

To ensure that our way of measuring trust is valid, we run additional robustness checks in which we employ different trust measures: In Supplementary Table 3, we provide different ways of encoding generalized social trust, both as different binary variables and an ordinal measure. In Supplementary Table 5, we extend our measure of social trust and use measures of trust towards different groups instead of the generalized social trust question. The results point in a similar direction: Social trust increases with conflict exposure towards family members, neighbors, Christians, Sunnis and Turkmens. A regression with an additive index of trust in all these groups as the dependent variable (column 9) implies that trust in people of different groups also significantly increases with conflict exposure. The only two groups for whom we do not find significantly positive effects are Shias and Alawites. This might be explained by the fact that these groups were most implicated in the fighting (Hall and Kahn, 2020).

Owing to the ease of interpretation, we have reported OLS results for all regressions on institutional trust in this article. In Supplementary Table 13, we refit our models using ordered logit regressions to show that our results are also robust to changes in the functional form.

All in all, our robustness checks make us confident that we have valid and highly robust results. While it is impossible to exogenously induce conflict exposure and thereby unfeasible to completely rule out endogeneity concerns in studies about conflict exposure (Bauer et al., 2016), our analysis makes it appear unlikely that self-selection or any omitted factors are the drivers of our results.

## Discussion

A topic that requires high degrees of intercultural cooperation, but is often neglected in the literature on cross-cultural cooperation, is the incorporation of migrants and refugees in the host country. Given the opportunities and risks that the arrival of high numbers of refugees brings about, it is a topic of utmost importance. Tragically, due to the most recent outflow of refugees from Ukraine, the topic has gained renewed relevance. A key prerequisite for cooperation between people in the host community and refugees is mutual trust. Additionally, the refugees’ trust in institutions in the host country is essential to enable them to actively participate in the society and economy.

The existing literature on trust often focuses on two potential sources of trust: The cultural background of migrants and the institutional context (see Dinesen, 2013 for an overview). The cultural perspective assumes trust to be rather stable over time and to be passed on from one generation to the next (e.g., Buchan et al., 2002; Uslaner, 2002, 2008; Sønderskov and Dinesen, 2016; Jamil and Baniamin, 2021; Karaja and Rubin, 2021). The institutional perspective, on the other hand, assumes that trust can change over time and focuses on experiences with high- or low-quality institutions in a country (e.g., Levi, 1996; Rothstein and Stolle, 2008; Freitag and Bühlmann, 2009). This trust literature sometimes uses flows of migration as a natural experiment to disentangle these two factors (e.g., Dinesen, 2013; Nannestad et al., 2014; Wu, 2020). We do not intend to focus on any of these factors but expand this literature by adding another variable that appears highly relevant for refugees’ propensity to trust: Exposure to conflict and potentially traumatic wartime events. The extremely unsettling experiences most refugees have lived through may have a lasting impact on their propensity to trust. Having observed people fighting each other and having experienced institutional breakdown are experiences that are likely to have affected both social and institutional trust.

We surveyed a large and diverse sample of Syrian and Iraqi refugees living in Turkey. We show that, in order to understand trust among refugees, it is indeed important to look beyond shared cultural background and the quality of institutions in their country of origin. Individual differences in exposure to different forms of violence and potentially traumatic events are important factors that should not be ignored.

Our results imply that conflict exposure is positively related to social trust. This finding is supportive of the growing literature that suggests conflict exposure has positive effects on various forms of social capital (e.g., Bauer et al., 2016). As most studies in this literature either implicitly or explicitly signaled to their subjects that they were interacting with in-group members, we extend these findings by showing positive effects even for generalized trust. Interestingly, our findings are independent of various personal or cultural characteristics and are qualitatively similar for Syrian and Iraqi refugees as well as for different religious or ethnic groups.

The positive impact we find is largely driven by very personal and emotional experiences, such as serious injury or the loss of a family member. As posttraumatic growth does not explain our findings, we conjecture that their driver must be experiential factors. People with a high degree of personal exposure are also likely to have had positive interpersonal experiences, such as solidarity and mutual support, which may have increased their general trust in other people. This conjecture is in line with arguments by Staub and Vollhardt (2008) who distinguish altruism born of suffering from posttraumatic growth: They argue that certain experiences, such as having received help in times of suffering, can induce prosocial behavior and caring for others, independent of one’s individual and self-referential posttraumatic growth.

Regarding institutional trust, our most notable finding is that exposure to violence may affect trust in different institutions differently. In line with the positive findings for social trust, we also find a positive impact on trust in order-keeping and policy-implementing institutions. This effect is largely driven by subjects who had experienced forced evacuation or various forms of hardship (including indiscriminate shelling or bombing as well as lack of shelter, nutrition, or medical care). Due to their experiences during the conflict, these subjects may have felt the strongest need for protection and subsequently may have also benefited most from the help provided by civilian police forces formed by rebels. On the downside, we find that conflict exposure is negatively related to trust in political institutions. These are likely to be the institutions the refugees perceived to have let them down most in their home countries. This negative effect is mainly driven by those who had very personal and emotional experiences with violence and can also be explained by symptoms of PTSD.

Intriguingly, subjects with high levels of PTSD symptoms do not exhibit significantly lower levels of social trust than people with lower levels of PTSD. This finding is fascinating, because PTSD is commonly described as a “defensive, fearful stance of the world” (McCann and Pearlman, 1990; Staub and Vollhardt, 2008). As there are often stereotypes about people suffering from trauma, many people might assume that refugees are likely to be severely traumatized and thus suffer from distrust toward everybody. This could pose a great challenge to their social adaptation. We show that this stereotype is likely to be unwarranted. Despite their trauma, they do not distrust other people in the settlement country.

Taken together, our findings imply that the fear that refugees might not be able to trust and cooperate due to their conflict exposure is unfounded. Exposure to potentially traumatic events does not necessarily lead to distrust. On the contrary, we show that people with strong exposure to conflict and very personal traumatizing experiences are more willing to trust other people. They are also more trusting of Turkish courts and the police. These types of trust are crucial for fruitful interaction and cooperation in their host countries. This is good news for integration policies. At the same time, refugees who suffer from PTSD have significantly lower institutional trust. A first step to support their propensity to acculturate might be to take care of their psychological health. They might need therapy and psychological treatment to overcome their symptoms of trauma and trust host country institutions more.

Our results also imply that it is important to pay attention to the types of experiences that migrants may have gone through. Some experiences have more significance for trust than others. Moreover, some experiences are negatively, and other experiences positively related to trust. Being aware of this could help decision-makers with limited resources target the right people and design interventions that are in line with their needs. For example, refugees with high levels of social trust might not need interventions designed to establish trust in host country citizens, but they might need interventions that help them to develop trust in political institutions in the host country.

A limitation of our study is that conflict exposure cannot be randomized by design. However, we use the tragic fact that violence took non-targeted forms such as barrel bombing, chemical weapons, and indiscriminate shelling with low precision. This indiscriminate violence enables us to employ a quasi-experimental approach to investigate the impact of conflict exposure on social and institutional trust. We further address the issue of self-selection by operationalizing conflict exposure in different ways and running numerous robustness checks, including an analysis of subsamples. While we cannot completely rule out issues of self-selection, our robustness checks make us confident that our results are reliable. Future studies could try to find instrumental variables to more clearly address causal effects. It could also be advantageous to include more details on the refugees’ family status, their satisfaction with institutions in the home country and other variables on their background in future studies to get a clearer picture of their motives for migration. A fruitful avenue for future research could be to elaborate on how the different types of experiences people had during the conflict lead to different trust levels, and which mechanisms can explain these relationships. Finding out in more detail which experiences are likely to make people more trusting would be helpful to policymakers to identify potential key players in integration programs and use them and their networks to reach out to other refugees. A related limitation of our study is that we do not distinguish between exposure to potentially traumatic events during the most recent civil wars and earlier experiences. Future research could disaggregate such effects. Another highly interesting research question would be to examine how the different types of experiences relate to symptoms of PTSD. It would be promising to more clearly disentangle objective experiences from psychological consequences to find out what distinguishes people who develop symptoms of PTSD from those who do not.

## Data Availability Statement

The raw data supporting the conclusions of this article will be made available by the authors, without undue reservation.

## Ethics Statement

The studies involving human participants were reviewed and approved by Uppsala Ethical Review Board (Etikprövningsnämden, EPN) Dnr 2016/189, May 2016. The patients/participants provided their written informed consent to participate in this study.

## Author Contributions

All authors listed have made a substantial, direct, and intellectual contribution to the work and approved it for publication.

## Funding

The field work was funded by the Swedish Research Council through grant no. 421-2014-1347. Theory development is based on work supported by the U. S. Army Research Office through the Minerva Initiative under grant number W911NF1810089.

## Conflict of Interest

The authors declare that the research was conducted in the absence of any commercial or financial relationships that could be construed as a potential conflict of interest.

## Publisher’s Note

All claims expressed in this article are solely those of the authors and do not necessarily represent those of their affiliated organizations, or those of the publisher, the editors and the reviewers. Any product that may be evaluated in this article, or claim that may be made by its manufacturer, is not guaranteed or endorsed by the publisher.

## Footnotes

^1^These different findings may in part be caused by the fact that most studies explicitly or implicitly look at particularized trust rather than generalized trust (Bauer et al., 2016; Mironova and Whitt, 2018). Moreover, different kinds of experiences may have different impacts on trust.

^2^Our identification strategy relies on the quasi-random nature of this indiscriminate shelling and bombing (or at least group-selective violence, not targeting individuals) and the resulting conflict exposure of the refugees. As we will show in **Figure 1**, 72% of our sample have experienced indiscriminate shelling or bombing. We will discuss our identification strategy and provide robustness checks in Section “Robustness Checks”. In the Supplementary Material, we show additional robustness checks, in which we run our analysis only for those subjects who have experienced indiscriminate shelling or bombing or were forcibly evacuated.

^3^Distributions of the answers are provided in **Table 2**.

^4^We have to stress here that the question asks about any such experience in their lives and not experiences that are specific to the most recent civil wars. The relationship we examine is a more general one, without a particular focus on these recent wars – even though it is plausible that most of the exposure indeed happened in these recent wars.

^5^In addition, we test this non-parametrically. A Mann–Whitney-Wilcoxon test indicates that those participants who exhibit higher levels of generalized social trust, i.e., those who believe that most people can be trusted, also have higher levels of exposure than those participants who are not as trusting (*z* = −2.053; *p* = 0.0401).

^6^If feeling safe is an important variable conditioning the relationship between war exposure and trust, then we might not be able to find such a positive relationship among exposed people who stayed in their country of origin. Yet, among our sample of interest, namely refugees who managed to arrive in Turkey, we can clearly state that higher degrees of conflict exposure are related to higher levels of generalized social trust.

^7^A table with the full set of controls, in which we gradually add more controls as a robustness check, can be found in Supplementary Table 4.

^8^We present OLS results here for ease of interpretation, but provide ordered logistic regressions as a robustness check in Supplementary Table 13.

^9^For parties, it is rather small in size and not robust to the inclusion of controls. The relationship between war exposure and trust in politicians was appropriately signed (negative) but not statistically significant in either regression.

^10^A potential reason could be the differing roles played by the Turkish government in Syria and Iraq. We can only speculate about such reasons, but exploring the extent to which host government behavior in armed conflict affects refugees’ trust in the host country could provide a fruitful avenue for future research.

^11^Hence, we only report the results in the Supplementary Material (Supplementary Table 16).
