# Supplementary material for: Trauma and Trust: How War Exposure Shapes Social and Institutional Trust Among Refugees
Source: Front Psychol. 2022 Aug 16;13:786838. doi: 10.3389/fpsyg.2022.786838 (PMC9426640; doi:10.3389/fpsyg.2022.786838)
Supplement: Supplementary file 1 [file Data_Sheet_1.pdf]

Supplementary Material

1 Psychological measures of exposure to conflict

Supplementary Figure 1: Histogram of index of exposure to conflict (according to Harvard Trauma Questionnaire)

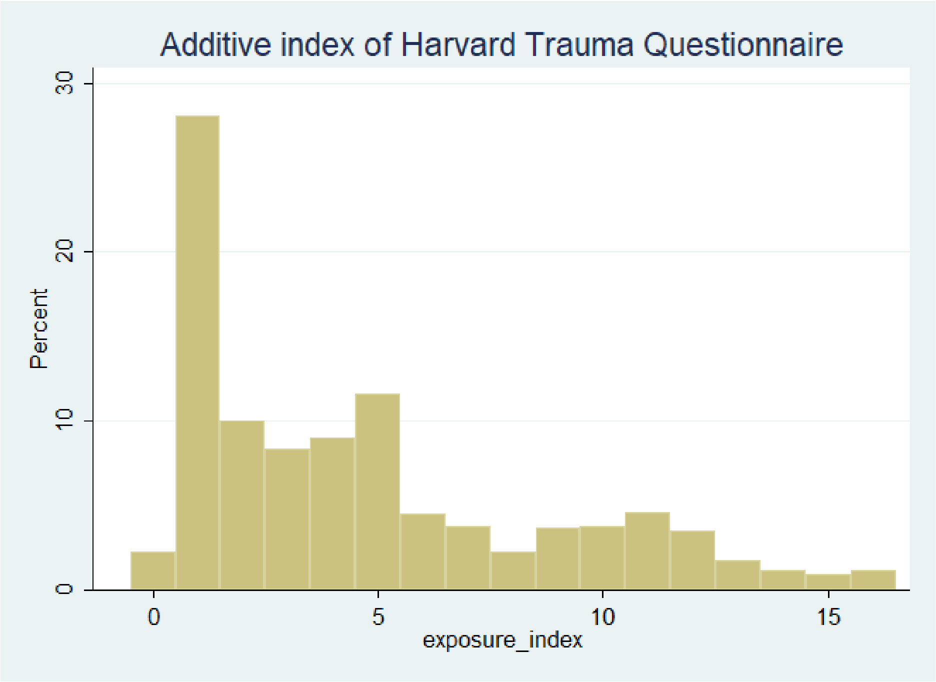

Supplementary Figure 2: Histogram of index of 6 symptoms of Posttraumatic Stress (according to civilian version of the PTSD Checklist (PCL-C)

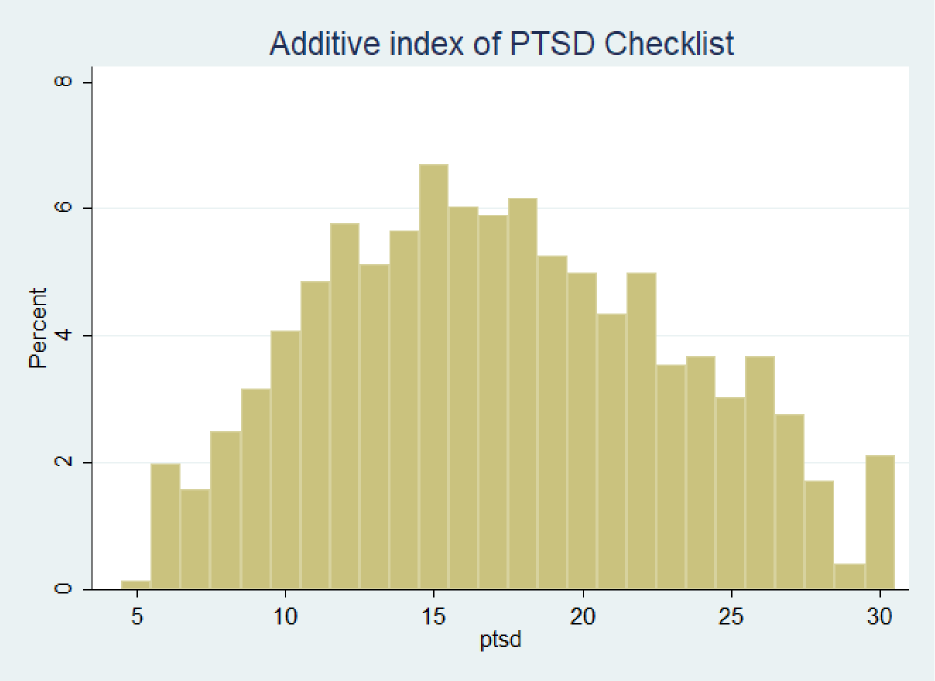

Note: In this index, a score of 14 or more is considered suggestive of difficulties with post-traumatic stress (Lang & Stein 2005). 70.9 percent of our sample fulfill this criterion.

Supplementary Figure 3: Histogram of index of Posttraumatic Growth (according to 10 items of Posttraumatic Growth Inventory-Short Form (PTGI-SF)

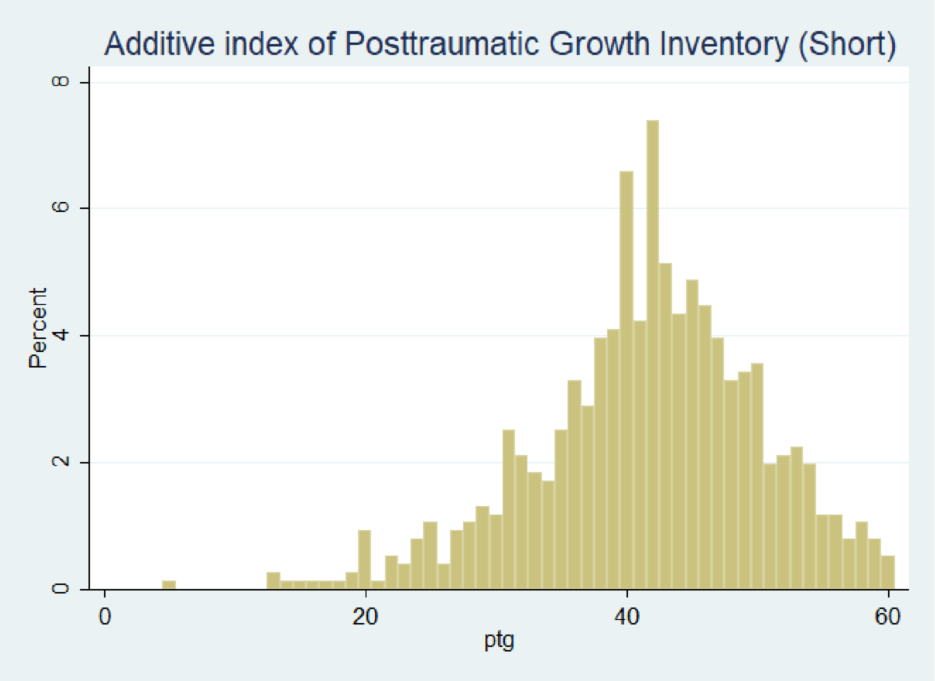

Supplementary Table 1: Correlations matrix of psychological measures

Variables	(1)	(2)	(3)
(1) exposure	1.000		
(2) ptsd	0.492*	1.000	
	(0.000)		
(3) ptg	0.110*	0.152*	1.000
	(0.002)	(0.000)	

p-values in parentheses

Supplementary Table 2: Principal Component Analysis, Rotated Components (oblique rotation)

Variable	Comp1	Comp2	Comp3	Comp4	Unexplained
Lack of food or water	0.0620	0.4843	-0.0252	-0.0092	.3185
Ill health w/out med. care	-0.0344	0.5157	-0.0659	0.0134	.3803
Lack of shelter	0.0203	0.4799	0.0170	0.0345	.3507
Imprisonment	0.2488	0.0037	0.2215	-0.0886	.5636
Physical abuse	0.1596	0.1494	0.2471	-0.0912	.5592
Serious injury	0.3371	0.1020	0.0626	-0.2226	.4313
Combat situation	0.3219	0.2109	0.0250	-0.3302	.3291
Indiscriminate shelling	-0.0769	0.3483	0.0585	0.1968	.6283

Being close to death	0.1787	-0.1315	0.0275	0.5904	.4041
Forced evacuation	-0.0767	0.2228	0.0165	0.5985	.4081
Forced separation from family	0.3516	-0.0335	0.0078	0.2134	.4095
Murder of family or friend	0.4260	-0.0606	-0.0816	0.1671	.3264
Unnatural death of fam./friend	0.4108	0.0053	-0.0435	0.0602	.3509
Murder of stranger(s)	0.4169	-0.0210	-0.0602	0.0229	.3944
Kidnapped	-0.0024	-0.0027	0.6484	-0.0117	.2295
Tortured	-0.0287	-0.0242	0.6668	0.0385	.2185

2 Robustness Checks on Social Trust

Supplementary Table 3: Exposure to Violence and Generalized Social Trust with different ways of encoding Generalized Social Trust and different model specifications

	(1)	(2)	(3)	(4)	(5)	(6)	(7)
	binary Logit	binary Logit	binary Logit	ordinal Logit	ordinal Logit	ordinal OLS	ordinal OLS
Exposure to Violence	0.02***	0.02***	0.01***	0.07***	0.07***	0.04***	0.05***
	(0.004)	(0.006)	(0.004)	(0.02)	(0.02)	(0.010)	(0.01)
**Demographics**							
female	-0.02	-0.07	-0.03	-0.1	-0.10	-0.08	-0.06
	(0.03)	(0.05)	(0.04)	(0.1)	(0.1)	(0.08)	(0.09)
age	0.003	0.008	-0.0001	0.008	0.004	0.005	0.006
	(0.01)	(0.02)	(0.01)	(0.05)	(0.05)	(0.03)	(0.03)
Syria	-0.10***	-0.2***	-0.03	-0.4***	-0.3**	-0.2***	-0.2**
	(0.04)	(0.05)	(0.04)	(0.1)	(0.1)	(0.09)	(0.09)
Social / economic status	-0.007	-0.03**	-0.02**	-0.06**	-0.05*	-0.04**	-0.03*
	(0.006)	(0.01)	(0.007)	(0.03)	(0.03)	(0.02)	(0.02)
Constant						1.9***	1.2***
						(0.1)	(0.1)
cut1							
Constant				-1.5***	-0.8***		
				(0.2)	(0.2)		
cut2							
Constant				-0.8***	0.9***		
				(0.2)	(0.2)		
cut3							
Constant				0.8***			
				(0.2)			
Observations	759	365	725	725	759	725	759
Pseudo R^2 / R^2	0.032	0.078	0.015	0.016	0.015	0.041	0.036

Robust standard errors in parentheses

* p<0.10, ** p<0.05, *** p<0.01

(1) - (3): Logistic regressions, marginal effects at means; (4) and (5): Ordered logistic regressions; (6) and (7): OLS

(1): Binary trust measure as used in main analysis

(2): Binary trust measure, 0= ”most cannot be trusted”, 1 = “most can be trusted”

(3): Binary trust measure, 0= “can’t be too careful” / “most cannot be trusted”, 1= ”most can be trusted” / “it depends”

(4), (6): Ordinal trust measure, 0 = “most cannot be trusted”, 1 = “can't be too careful”, 2 = “it depends”, 3 = “most can be trusted”

(5), (7): Ordinal trust measure, 0 = “most cannot be trusted” / ” can't be too careful”, 1 = “don't know” / “it depends”, 2 = “most can be trusted”

Supplementary Table 4: Exposure to Violence and Generalized Social Trust with successive inclusion of control variables.

	(1)	(2)	(3)	(4)	(5)	(6)	(7)	(8)
	Dep. Variable: Generalized social trust							
	Marginal effects at means							
Exposure to violence	0.02***	0.02***	0.02***	0.02***	0.02***	0.03***	0.03***	0.03***
	(0.004)	(0.004)	(0.004)	(0.005)	(0.005)	(0.005)	(0.005)	(0.005)
**Demographics**								
female		-0.01	-0.02	-0.03	-0.02	-0.03	-0.04	-0.03
		(0.03)	(0.03)	(0.04)	(0.04)	(0.04)	(0.04)	(0.04)
age		0.007	0.005	0.009	0.01	0.01	0.008	0.01
		(0.01)	(0.01)	(0.01)	(0.01)	(0.01)	(0.01)	(0.01)
Syria		-0.1***	-0.10***	-0.1***	-0.1***	-0.1**	-0.10**	-0.1***
		(0.04)	(0.04)	(0.04)	(0.04)	(0.05)	(0.05)	(0.05)
Social / economic status			-0.007	-0.004	-0.003	-0.006	-0.008	-0.007
			(0.006)	(0.007)	(0.007)	(0.007)	(0.007)	(0.007)
**Education**								
< 6 years of schooling			0.02	0.1	0.1	0.2	0.2	0.2
			(0.2)	(0.2)	(0.2)	(0.2)	(0.2)	(0.2)
6 years of schooling			-0.04	-0.09	-0.08	-0.09	-0.08	-0.08
			(0.1)	(0.2)	(0.2)	(0.2)	(0.2)	(0.2)
9 years of schooling			-0.06	-0.07	-0.07	-0.06	-0.04	-0.06
			(0.1)	(0.1)	(0.1)	(0.2)	(0.2)	(0.2)
12 years of schooling			-0.008	-0.01	-0.01	0.010	0.02	0.001
			(0.1)	(0.1)	(0.1)	(0.2)	(0.2)	(0.2)
> 12 years of schooling			-0.05	-0.06	-0.06	-0.03	-0.01	-0.02
			(0.1)	(0.1)	(0.1)	(0.2)	(0.2)	(0.2)
urban				-0.002	-0.02	-0.02	-0.01	-0.03
				(0.05)	(0.06)	(0.06)	(0.06)	(0.06)
Arrived in 2016				0.2***	0.2***	0.2***	0.2***	0.2***
				(0.05)	(0.05)	(0.05)	(0.05)	(0.05)

**Prospects of moving back**					
In 0-4 years	0.1**	0.1**	0.1*	0.1**	0.1**
	(0.05)	(0.05)	(0.05)	(0.05)	(0.05)
In 5-10 years	0.04	0.04	0.02	0.03	0.02
	(0.04)	(0.04)	(0.04)	(0.04)	(0.04)
When I am old.	0.08	0.09	0.09	0.08	0.08
	(0.06)	(0.06)	(0.07)	(0.07)	(0.07)
**Legal status**					
Citizenship		0.1	0.2	0.2	0.2
		(0.1)	(0.2)	(0.2)	(0.2)
Permanent protection		0.1	0.1	0.10	0.09
		(0.08)	(0.09)	(0.09)	(0.09)
Temporary protection		0.02	0.002	0.005	0.001
		(0.06)	(0.07)	(0.07)	(0.07)
Temporary suspension of deportation		-0.1	-0.1	-0.1	-0.2
		(0.2)	(0.2)	(0.2)	(0.2)
Asylum seeker		-0.04	-0.03	-0.02	-0.03
		(0.06)	(0.06)	(0.06)	(0.07)
No doc. / waiting		-0.1	-0.1	-0.1	-0.01
		(0.1)	(0.1)	(0.1)	(0.1)
**Political attitude**					
Social dominance orientation			0.006	0.004	0.003
			(0.009)	(0.009)	(0.009)
Right-wing authoritarianism			0.009	0.010	0.008
			(0.010)	(0.010)	(0.01)
**Religion**					
Muslim Sunni				-0.2*	-0.5***
				(0.1)	(0.1)
Muslim Shia				0.05	-0.3*
				(0.2)	(0.2)
Other				-0.2	-0.5***

							(0.2)	(0.2)
Yazidi							-0.2	
							(0.3)	
**Ethnicity**								
Kurd								-0.1
								(0.1)
Turkman								-0.09
								(0.07)
Assyrian								-0.3***
								(0.02)
Armenian								-0.2***
								(0.07)
Observations	791	765	758	726	722	680	677	659
Pseudo R^2	0.016	0.031	0.034	0.054	0.062	0.074	0.083	0.093

Logistic regressions, Marginal effects at means, Robust standard errors in parentheses

* p<0.10, ** p<0.05, *** p<0.01

Dependent variable: Social Trust (binary measure, 0 = “most cannot be trusted” / “can't be too careful” / “it depends” / “I don’t know”; 1 = “most people can be trusted”)

Independent variables:

Exposure: Additive index of the 16-item Harvard Trauma Questionnaire

Female: dummy variable indicating respondent’s gender; 0 = male, 1 = female

Age: proxy of age; 1 = 18-24, 2 = 25-24, 3 = 35-44, 4 = 45-54, 5 = 55-64, 6 = 65-74, 7 = 75-84, 8 = 85 or older

Syria: dummy variable indicating respondent’s country of origin; 0 = Iraq, 1 = Syria

Further controls:

Social / economic status: Self-reported status in country of origin, 0 (the worst off) … 10 (the best off)

Education: Indicator variable, 1 = no formal education, 2 = < 6 years of schooling, 3 = 6 years of schooling, 4 = 9 years of schooling, 5 = 12 years of schooling, 6 = > 12 years of schooling

Urban: Dummy variable indicating whether a respondent self-reported to come from a rural (0) or urban (1) region

Return: Indicator variable on belief to move back; 0 = No, I do not think I will move back; 1 = Yes, 0 to 4 years from now; 2 = Yes, 5 to 10 years from now; 3 = Yes, when I am old.

Legal status: Indicator variable on current legal status in Turkey, 1 = Turkish citizen, 1 = Permanent protection, 3 = Temporary protection, 4 = Temporary suspension of deportation, 5 = Asylum seeker, 6 = No documentation or waiting for documentation, 7 = Denied protection, 8 = Other

Social dominance orientation: Additive index of 6 items of Social Dominance Orientation Scale

Right-wing authoritarianism: Additive index of 3 items of Right-Wing Authoritarianism Scale

Supplementary Table 5: Exposure to Violence and Social Trust towards certain groups

	(1)<br>family	(2)<br>neighbors	(3)<br>Christians	(4)<br>Kurds	(5)<br>Shia	(6)<br>Alawi	(7)<br>Sunni	(8)<br>Turkoman	(9)<br>index
conflict exposure	0.009**	0.02***	0.02***	0.02***	0.0005	0.002	0.01*	0.03***	0.1***
	(0.004)	(0.005)	(0.005)	(0.004)	(0.002)	(0.002)	(0.005)	(0.005)	(0.02)
**Demographics**									
female	-0.005	0.01	-0.05	-0.003	-0.03	-0.03	-0.03	-0.06	-0.1
	(0.03)	(0.04)	(0.04)	(0.03)	(0.02)	(0.02)	(0.04)	(0.04)	(0.1)
age	0.009	0.02	0.05***	-0.003	0.002	-0.002	0.02	-0.02	0.1**
	(0.01)	(0.01)	(0.01)	(0.01)	(0.005)	(0.005)	(0.02)	(0.01)	(0.05)
Syria	-0.05	-0.05	-0.2***	-0.10***	-0.06***	-0.02	-0.2***	-0.08*	-0.8***
	(0.03)	(0.05)	(0.05)	(0.04)	(0.02)	(0.02)	(0.05)	(0.04)	(0.2)
Social / economic status	-0.01*	-0.004	0.02**	0.003	0.004	0.003	-0.04***	-0.01	-0.06**
	(0.006)	(0.008)	(0.008)	(0.006)	(0.003)	(0.003)	(0.008)	(0.008)	(0.03)
urban	-0.03	-0.06	-0.09	-0.1**	-0.02	-0.02	-0.1*	-0.05	-0.5**
	(0.04)	(0.06)	(0.06)	(0.04)	(0.03)	(0.02)	(0.07)	(0.06)	(0.2)
Arrived in 2016	0.03	0.2***	0.1**	0.02	0.006	-0.0003	0.2***	0.08	0.7***
	(0.04)	(0.06)	(0.05)	(0.04)	(0.02)	(0.02)	(0.07)	(0.05)	(0.2)
**Education**									
< 6 years of schooling	0.2	-0.1	0.10	-0.06	-0.05	0.02	0.05	-0.3*	0.08
	(0.2)	(0.2)	(0.2)	(0.10)	(0.1)	(0.1)	(0.2)	(0.1)	(0.8)
6 years of schooling	0.3*	0.04	0.04	0.04	0.009	0.03	0.1	-0.03	0.7
	(0.1)	(0.1)	(0.1)	(0.1)	(0.1)	(0.09)	(0.2)	(0.2)	(0.6)
9 years of schooling	0.4***	0.02	-0.04	-0.006	-0.09	-0.05	0.2*	0.03	0.7
	(0.1)	(0.1)	(0.1)	(0.09)	(0.10)	(0.07)	(0.1)	(0.1)	(0.5)
12 years of schooling	0.3**	-0.03	0.06	0.03	-0.09	-0.03	0.10	-0.05	0.5
	(0.1)	(0.1)	(0.1)	(0.09)	(0.10)	(0.07)	(0.1)	(0.1)	(0.5)
> 12 years of schooling	0.4***	-0.01	0.1	0.08	-0.07	-0.03	0.2	-0.01	0.8
	(0.1)	(0.1)	(0.1)	(0.08)	(0.10)	(0.07)	(0.1)	(0.1)	(0.5)
**Prospects**									

	(1)	(2)	(3)	(4)	(5)	(6)	(7)	(8)	(9)
**of moving back**									
0 to 4 years from now.	0.1***	0.09*	-0.06	0.01	0.008	0.02	0.1***	0.03	0.2
	(0.04)	(0.05)	(0.05)	(0.04)	(0.03)	(0.02)	(0.06)	(0.05)	(0.2)
Yes, 5 to 10 years from now.	0.09**	0.03	-0.08	-0.02	-0.01	0.007	0.06	-0.002	0.02
	(0.04)	(0.05)	(0.05)	(0.03)	(0.02)	(0.02)	(0.05)	(0.05)	(0.2)
Yes, when I am old.	0.02	-0.05	-0.01	-0.02	-0.01	-0.010	-0.04	-0.1**	-0.3
	(0.05)	(0.07)	(0.07)	(0.05)	(0.03)	(0.02)	(0.07)	(0.06)	(0.3)
N	724	713	709	712	708	706	710	716	694
pseudo R^2	0.076	0.048	0.093	0.070	0.053	0.048	0.107	0.075	0.040

Logistic regressions (column (1)-(8)) / ordered logistic regression (column (9)), robust standard errors in parentheses
* p<0.10, ** p<0.05, *** p<0.01

Dependent variables: Trust in members of various groups; , 0 = “most cannot be trusted” / “can't be too careful” / “it depends” / “I don’t know”; 1 = “most can be trusted”

(1) Members of your family
(2) People in your current neighborhood
(3) Christians
(4) Kurds
(5) Shia Arabs
(6) Alawi
(7) Sunni Arabs
(8) Turkomans
(9) Additive index of all the binary measures

Independent variables:

Exposure: Additive index of the 16-item Harvard Trauma Questionnaire

Female: dummy variable indicating respondent’s gender; 0 = male, 1 = female

Age: proxy of age; 1 = 18-24, 2 = 25-34, 3 = 35-44, 4 = 45-54, 5 = 55-64, 6 = 65-74, 7 = 75-84, 8 = 85 or older

Syria: dummy variable indicating respondent’s country of origin; 0 = Iraq, 1 = Syria

Social / economic status: Self-reported status in country of origin, 0 (the worst off) … 10 (the best off)

Urban: Dummy variable indicating whether a respondent self-reported to come from a rural (0) or urban (1) region

Arrived in 2016: Dummy variable indicating whether a respondent arrived in Turkey in 2016 (1) or not (0) region

Further controls:

Education: Indicator variable, 1 = no formal education, 2 = < 6 years of schooling, 3 = 6 years of schooling, 4 = 9 years of schooling, 5 = 12 years of schooling, 6 = > 12 years of schooling

Return: Indicator variable on belief to move back; 0 = No, I do not think I will move back; 1 = Yes, 0 to 4 years from now; 2 = Yes, 5 to 10 years from now; 3 = Yes, when I am old.

Supplementary Table 6: Regressions of Generalized Social Trust on Exposure to violence at a level of Subsamples

6a: Subsamples for which exposure is arguably less systematic

	(1)	(2)	(3)	(4)
	Indisc. shelling	Forced evac.	<=24	<= 24, arrived before 2016
conflict exposure	0.026***	0.027***	0.014**	0.015**
	(0.006)	(0.007)	(0.006)	(0.006)
**Demograph.**				
female	0.0091	0.057	-0.026	-0.0064
	(0.04)	(0.05)	(0.05)	(0.05)
age	0.0023	0.016		
	(0.01)	(0.02)		
Syria	-0.10**	-0.21***	-0.14**	-0.18***
	(0.05)	(0.07)	(0.06)	(0.06)
Social / econ. status	-0.0052	0.000034	-0.022**	-0.019**
	(0.009)	(0.010)	(0.009)	(0.009)
urban	-0.024	-0.13	-0.014	-0.043
	(0.07)	(0.09)	(0.07)	(0.07)
Arrived in 2016	0.16***	0.068	0.055	
	(0.06)	(0.08)	(0.08)	
**Education**				
< 6 years of schooling	0.026	0.20	-0.18	-0.17
	(0.2)	(0.3)	(0.3)	(0.3)
6 years of schooling	-0.13	-0.20	-0.24	-0.22
	(0.2)	(0.2)	(0.2)	(0.2)
9 years of schooling	-0.16	-0.028	-0.18	-0.17
	(0.2)	(0.2)	(0.2)	(0.2)
12 years of schooling	-0.081	0.089	-0.12	-0.13
	(0.2)	(0.2)	(0.2)	(0.2)
> 12 years of schooling	-0.12	0.063	-0.18	-0.15
	(0.2)	(0.2)	(0.2)	(0.2)
**Prospects of**				

**moving back**				
0 to 4 years from now.	0.059	0.052	0.15**	0.17***
	(0.06)	(0.07)	(0.06)	(0.06)
5 to 10 years from now.	0.023	-0.022	0.11**	0.10*
	(0.05)	(0.06)	(0.06)	(0.05)
When I am old.	0.068	0.092	0.065	0.091
	(0.09)	(0.1)	(0.08)	(0.08)
Obs.	537	389	265	235
Pseudo R^2	0.051	0.075	0.120	0.137

6b: Other prominent subsamples

	(5) Syria	(6) Iraq	(7) Sunni	(8) non-Sunni	(9) Arab	(10) non-Arab	(11) male	(12) female
conflict exposure	0.02***	0.02***	0.02***	0.01	0.02***	0.02*	0.02***	0.03***
	(0.006)	(0.007)	(0.005)	(0.01)	(0.005)	(0.01)	(0.006)	(0.008)
**Demograph.**								
female	-0.02	-0.02	-0.02	-0.09	-0.03	-0.01		
	(0.05)	(0.05)	(0.04)	(0.1)	(0.04)	(0.06)		
age	0.02	-0.008	0.01	-0.06	0.02	-0.06**	0.01	0.003
	(0.02)	(0.02)	(0.01)	(0.05)	(0.01)	(0.03)	(0.02)	(0.02)
Syria			-0.09**	-0.4**	-0.1**	-0.3***	-0.1*	-0.1*
			(0.04)	(0.2)	(0.05)	(0.09)	(0.06)	(0.07)
Social / econ. status	-0.02*	0.007	-0.009	0.005	-0.0005	-0.03**	0.003	-0.02
	(0.01)	(0.009)	(0.007)	(0.03)	(0.008)	(0.01)	(0.009)	(0.01)
urban	-0.01	0.2	-0.006	-0.08	-0.01	0.03	-0.01	-0.01
	(0.06)	(0.1)	(0.06)	(0.3)	(0.06)	(0.10)	(0.07)	(0.08)
Arrived in 2016	0.1*	0.1**	0.1***	0.7	0.2***	0.1	0.2***	0.1*
	(0.07)	(0.07)	(0.05)	(0.4)	(0.05)	(0.1)	(0.07)	(0.07)
**Education**								
< 6 years of schooling	0.2	0.1	-0.01	0	0.3	-0.06	0	-0.2
	(0.3)	(0.2)	(0.2)	(.)	(0.2)	(0.10)	(.)	(0.2)
6 years of	-0.09	0.03	-0.2	0	-0.08	0.01	0	-0.4**

schooling	(0.2)	(0.2)	(0.2)	(.)	(0.2)	(0.1)	(.)	(0.2)
9 years of schooling	-0.09	0.09	-0.2	0	-0.06	0.08	0	-0.4*
	(0.2)	(0.2)	(0.2)	(.)	(0.2)	(0.2)	(.)	(0.2)
12 years of schooling	-0.07	0.2	-0.2	0	0.03	-0.04	0	-0.3*
	(0.2)	(0.2)	(0.2)	(.)	(0.2)	(0.1)	(.)	(0.2)
> 12 years of schooling	-0.1	0.1	-0.2	0	-0.04	0.07	0	-0.4**
	(0.2)	(0.2)	(0.2)	(.)	(0.2)	(0.09)	(.)	(0.2)
**Prospects of moving back**								
0 to 4 years from now.	0.08	0.1*	0.1***	0.09	0.09	0.2**	0.06	0.2**
	(0.07)	(0.06)	(0.05)	(0.2)	(0.05)	(0.07)	(0.06)	(0.08)
5 to 10 years from now.	-0.06	0.09*	0.05	0.09	-0.0004	0.2*	0.05	0.03
	(0.06)	(0.05)	(0.04)	(0.2)	(0.05)	(0.1)	(0.06)	(0.06)
When I am old.	-0.05	0.2**	0.08	0.04	0.04	0.2*	-0.01	0.3**
	(0.08)	(0.09)	(0.07)	(0.2)	(0.07)	(0.1)	(0.08)	(0.1)
Obs.	288	438	646	76	616	110	435	284
Pseudo R^2	0.102	0.047	0.062	0.143	0.054	0.291	0.048	0.107

Logistic regressions, marginal effects at means, robust standard errors in parentheses

* p<0.10, ** p<0.05, *** p<0.01

Dependent variable: Social Trust (binary measure, 0 = “most cannot be trusted” / “can't be too careful” / “it depends” / “I don’t know”; 1 = “most people can be trusted”)

(1) Restricted to those who reported to have experienced indiscriminate shelling or bombing

(2) Restricted to those who reported to have been forcibly evacuated

(3) Restricted to youngest subsample (aged 24 or younger)

(4) Restricted to youngest during war (aged 24 or younger and arrived in Turkey before 2016)

(5) Restricted to refugees from Syria

(6) Restricted to refugees from Iraq

(7) Restricted to Sunni Muslims

(8) Restricted to all other religious groups except Sunni

(9) Restricted to subjects of Arab ethnicity

(10) Restricted to all other ethnic groups except Arab

(11) Restricted to males

(12) Restricted to females

Independent variables:

Exposure: Additive index of the 16-item Harvard Trauma Questionnaire

Female: dummy variable indicating respondent’s gender; 0 = male, 1 = female

Age: proxy of age; 1 = 18-24, 2 = 25-34, 3 = 35-44, 4 = 45-54, 5 = 55-64, 6 = 65-74, 7 = 75-84, 8 = 85 or older

Syria: dummy variable indicating respondent’s country of origin; 0 = Iraq, 1 = Syria

Social / economic status: Self-reported status in country of origin, 0 (the worst off) … 10 (the best off)

Urban: Dummy variable indicating whether a respondent self-reported to come from a rural (0) or urban (1) region

Arrived in 2016: Dummy variable indicating whether a respondent arrived in Turkey in 2016 (1) or not (0) region

Further controls:

Education: Indicator variable, 1 = no formal education, 2 = < 6 years of schooling, 3 = 6 years of schooling, 4 = 9 years of schooling, 5 = 12 years of schooling, 6 = > 12 years of schooling

Return: Indicator variable on belief to move back; 0 = No, I do not think I will move back; 1 = Yes, 0 to 4 years from now; 2 = Yes, 5 to 10 years from now; 3 = Yes, when I am old.

For non-Sunnis and males, the education dummies had to be dropped due to multicollinearity.

6c: Wald tests for equality of coefficient for conflict exposure

	Indisc. Shelling = 1 vs. 0	Forced evac. = 1 vs. 0	Syria vs. Iraq	Sunni vs. non-Sunni	Arab vs. non-Arab	Male vs. female
Chi^2	1.62	0.08	0.06	1.07	1.59	4.35**
p	0.020	0.77	0.81	0.30	0.21	0.04

Wald-tests for the equality of coefficients between pairs of subsamples detect no significant differences between the coefficient for exposure, except between male and female refugees, where the effect is significantly larger for females.

Supplementary Table 7: Regressions of Generalized Social Trust on Exposure to violence interacted with employment status

	(1)
Exposure to violence	0.06*
	(0.03)
retired, student or housewife	0.3
	(0.3)
manual / craftsperson	0.03
	(0.4)
high-ranked professions	-0.7
	(1.2)
retired, student or housewife # exposure	-0.1*
	(0.06)
manual / craftsperson # exposure	0.09*
	(0.05)
high-ranked professions # exposure	0.3**
	(0.1)
Constant	-1.3***
	(0.2)
Observations	783
Pseudo R^2	0.049

Logistic regressions, robust standard errors in parentheses
* p<0.10, ** p<0.05, *** p<0.01
Dependent variable: Social Trust (binary measure, 0 = “most cannot be trusted” / “can't be too careful” / “it depends” / “I don’t know”; 1 = “most people can be trusted”)
Independent variables:
Exposure: Additive index of the 16-item Harvard Trauma Questionnaire
Self-reported employment status:
Omitted reference category: Unemployed
Retired, student or housewife
Manual / craftsperson: Manual laborer, Agricultural worker, Member of the armed forces / security, Owner of a shop, Private sector employee, Craftsperson, Other
High-ranked professions: Employer / director of an institution with less than 10 employees, Employer / director of an institution with 10 employees or more, Professional such as lawyer, accountant, teacher, doctor, etc, Government employee

3 Robustness Checks on Institutional Trust

Supplementary Table 8: Exposure to Violence and Institutional Trust with demographic controls.

	(1) Courts	(2) Police	(3) Politicians	(4) Parties	(5) Parliament	(6) Government
Exposure to violence	0.021**	0.043***	-0.013	-0.015	-0.074***	-0.073***
	(0.009)	(0.009)	(0.01)	(0.01)	(0.01)	(0.01)
**Demographics**						
female	-0.13*	-0.077	-0.0085	0.063	-0.24***	-0.24***
	(0.07)	(0.08)	(0.1)	(0.10)	(0.09)	(0.08)
age	0.014	-0.019	-0.020	-0.067*	0.033	0.029
	(0.03)	(0.03)	(0.03)	(0.04)	(0.03)	(0.03)
Syria	-0.33***	-0.18**	-0.63***	-0.51***	-0.24***	-0.24***
	(0.07)	(0.08)	(0.1)	(0.1)	(0.09)	(0.09)
Social / economic status	-0.059***	-0.050***	-0.025	-0.082***	-0.085***	-0.10***
	(0.01)	(0.01)	(0.02)	(0.02)	(0.02)	(0.02)
Constant	4.50***	4.38***	3.89***	4.02***	4.54***	4.84***
	(0.1)	(0.1)	(0.2)	(0.2)	(0.1)	(0.1)
Observations	744	741	739	740	742	740
R^2	0.069	0.047	0.057	0.067	0.126	0.155

OLS regressions, robust standard errors in parentheses

* p<0.10, ** p<0.05, *** p<0.01

Dependent variables: Self-reported trust in institutions in Turkey, 1 = “Do not trust at all”, 2 = “Do not trust very much”, 3 = “I don’t know”, 4 = “Trust somewhat”, 5 = “Trust completely”

Independent variables:

Exposure: Additive index of the 16-item Harvard Trauma Questionnaire

Female: dummy variable indicating respondent’s gender; 0 = male, 1 = female

Age: proxy of age; 1 = 18-24, 2 = 25-34, 3 = 35-44, 4 = 45-54, 5 = 55-64, 6 = 65-74, 7 = 75-84, 8 = 85 or older

Syria: dummy variable indicating respondent’s country of origin; 0 = Iraq, 1 = Syria

Social / economic status: Self-reported status in country of origin, 0 (the worst off) … 10 (the best off)

Supplementary Table 9: Exposure to Violence and Institutional Trust (Table 4) with all coefficients.

	(1)<br>Courts	(2)<br>Police	(3)<br>Politicians	(4)<br>Parties	(5)<br>Parliament	(6)<br>Government
exposure to violence	0.027***	0.042***	-0.017	-0.019	-0.069***	-0.070***
	(0.010)	(0.01)	(0.01)	(0.01)	(0.01)	(0.01)
**Demographics**						
female	-0.14*	-0.050	0.0062	0.064	-0.25***	-0.25***
	(0.08)	(0.08)	(0.10)	(0.10)	(0.09)	(0.08)
age	0.029	-0.010	-0.014	-0.069*	0.039	0.042
	(0.03)	(0.03)	(0.04)	(0.04)	(0.03)	(0.03)
Syria	-0.34***	-0.090	-0.57***	-0.47***	-0.20*	-0.21**
	(0.09)	(0.10)	(0.1)	(0.1)	(0.1)	(0.1)
Social / economic status	-0.050***	-0.041***	-0.0025	-0.065***	-0.078***	-0.093***
	(0.01)	(0.02)	(0.02)	(0.02)	(0.02)	(0.02)
urban	0.000037	0.096	0.017	0.0083	0.14	0.094
	(0.1)	(0.1)	(0.1)	(0.1)	(0.1)	(0.1)
Arrived in 2016	0.28***	0.031	0.11	0.094	0.10	0.42***
	(0.1)	(0.1)	(0.1)	(0.1)	(0.1)	(0.1)
**Education**						
< 6 years of schooling	0.20	-0.15	-0.21	-0.081	-0.24	0.82*
	(0.3)	(0.4)	(0.4)	(0.4)	(0.5)	(0.5)
6 years of schooling	-0.12	0.26	0.56*	0.51*	0.0097	0.41
	(0.2)	(0.3)	(0.3)	(0.3)	(0.3)	(0.4)
9 years of schooling	0.18	0.76***	0.93***	1.07***	0.39	0.93***
	(0.2)	(0.3)	(0.3)	(0.3)	(0.3)	(0.4)
12 years of schooling	0.097	0.65**	0.73**	0.68**	0.17	0.79**
	(0.2)	(0.3)	(0.3)	(0.3)	(0.3)	(0.4)
> 12 years of schooling	0.0017	0.44*	0.48*	0.53**	0.084	0.71**
	(0.2)	(0.3)	(0.3)	(0.3)	(0.3)	(0.3)
**Prospects of moving back**						
0 to 4 years from now.	0.26**	0.25**	0.70***	0.81***	0.54***	0.42***
	(0.1)	(0.1)	(0.1)	(0.1)	(0.1)	(0.1)

Yes, 5 to 10 years from now.	0.17*	0.12	0.23*	0.24*	0.29***	0.25**
	(0.10)	(0.1)	(0.1)	(0.1)	(0.1)	(0.1)
Yes, when I am old.	0.15	0.017	-0.52***	-0.50***	0.085	0.23
	(0.1)	(0.1)	(0.2)	(0.2)	(0.2)	(0.1)
Constant	4.17***	3.58***	2.96***	3.09***	3.95***	3.65***
	(0.2)	(0.3)	(0.3)	(0.3)	(0.3)	(0.4)
Observations	713	710	709	711	712	710
R^2	0.097	0.090	0.171	0.204	0.182	0.208

OLS regressions, robust standard errors in parentheses

* p<0.10, ** p<0.05, *** p<0.01

Dependent variables: Self-reported trust in institutions in Turkey, 1 = “Do not trust at all”, 2 = “Do not trust very much”, 3 = “I don’t know”, 4 = “Trust somewhat”, 5 = “Trust completely”

Independent variables:

Exposure: Additive index of the 16-item Harvard Trauma Questionnaire

Female: dummy variable indicating respondent’s gender; 0 = male, 1 = female

Age: proxy of age; 1 = 18-24, 2 = 25-34, 3 = 35-44, 4 = 45-54, 5 = 55-64, 6 = 65-74, 7 = 75-84, 8 = 85 or older

Syria: dummy variable indicating respondent’s country of origin; 0 = Iraq, 1 = Syria

Social / economic status: Self-reported status in country of origin, 0 (the worst off) … 10 (the best off)

Urban: Dummy variable indicating whether a respondent self-reported to come from a rural (0) or urban (1) region

Arrived in 2016: Dummy variable indicating whether a respondent arrived in Turkey in 2016 (1) or not (0) region

Further controls:

Education: Indicator variable, 1 = no formal education, 2 = < 6 years of schooling, 3 = 6 years of schooling, 4 = 9 years of schooling, 5 = 12 years of schooling, 6 = > 12 years of schooling

Return: Indicator variable on belief to move back; 0 = No, I do not think I will move back; 1 = Yes, 0 to 4 years from now; 2 = Yes, 5 to 10 years from now; 3 = Yes, when I am old.

Supplementary Table 10: Exposure to Violence and Institutional Trust with full set of control variables.

	(1)<br>Courts	(2)<br>Police	(3)<br>Politicians	(4)<br>Parties	(5)<br>Parliament	(6)<br>Government
exposure to violence	0.017	0.039***	-0.023	-0.025*	-0.074***	-0.071***
	(0.01)	(0.01)	(0.01)	(0.01)	(0.01)	(0.01)
**Demographics**						
female	-0.093	-0.051	0.040	0.069	-0.21**	-0.19**
	(0.07)	(0.08)	(0.1)	(0.10)	(0.09)	(0.08)
age	0.023	-0.022	-0.012	-0.081**	0.036	0.053*
	(0.03)	(0.03)	(0.04)	(0.04)	(0.03)	(0.03)
Syria	-0.43***	-0.14	-0.62***	-0.52***	-0.28**	-0.24**
	(0.10)	(0.1)	(0.1)	(0.1)	(0.1)	(0.1)
Social / economic status	-0.047***	-0.032**	0.0096	-0.055***	-0.067***	-0.087***
	(0.01)	(0.02)	(0.02)	(0.02)	(0.02)	(0.02)
urban	-0.10	0.026	0.0058	-0.0039	0.060	0.062
	(0.1)	(0.1)	(0.1)	(0.1)	(0.1)	(0.1)
Arrived in 2016	0.32***	0.070	0.16	0.16	0.14	0.48***
	(0.1)	(0.1)	(0.1)	(0.2)	(0.1)	(0.1)
**Education**						
< 6 years of schooling	0.016	-0.44	-0.43	-0.26	-0.50	0.57
	(0.3)	(0.4)	(0.4)	(0.4)	(0.5)	(0.5)
6 years of schooling	-0.024	0.24	0.61*	0.63**	-0.037	0.53
	(0.2)	(0.3)	(0.4)	(0.3)	(0.4)	(0.4)
9 years of schooling	0.090	0.60**	1.00***	1.09***	0.24	0.85**
	(0.2)	(0.3)	(0.3)	(0.3)	(0.3)	(0.4)
12 years of schooling	0.0077	0.49*	0.80**	0.78**	0.035	0.75**
	(0.2)	(0.3)	(0.3)	(0.3)	(0.3)	(0.4)
> 12 years of schooling	-0.034	0.35	0.58*	0.66**	0.023	0.72**
	(0.2)	(0.3)	(0.3)	(0.3)	(0.3)	(0.3)
**Prospects of moving back**						
0 to 4 years from now.	0.10	0.091	0.54***	0.58***	0.36***	0.34***
	(0.1)	(0.1)	(0.1)	(0.1)	(0.1)	(0.1)

Yes, 5 to 10 years from now.	0.082	0.046	0.17	0.16	0.22*	0.24**
	(0.10)	(0.1)	(0.1)	(0.1)	(0.1)	(0.1)
Yes, when I am old.	0.12	0.019	-0.58***	-0.52***	0.090	0.23
	(0.1)	(0.1)	(0.2)	(0.2)	(0.2)	(0.1)
**Legal status**						
Citizenship	0.58**	-0.84	0.14	-0.38	-0.14	-0.054
	(0.2)	(0.5)	(0.5)	(0.5)	(0.5)	(0.3)
Permanent protection	0.33*	0.10	-0.16	-0.34*	0.17	-0.060
	(0.2)	(0.2)	(0.2)	(0.2)	(0.2)	(0.2)
Temporary protection	0.40***	0.29**	0.11	0.22	0.27**	0.11
	(0.1)	(0.1)	(0.2)	(0.1)	(0.1)	(0.1)
Temporary suspension of deportation	0.96***	0.80***	1.55***	1.86***	1.25***	1.17***
	(0.2)	(0.3)	(0.3)	(0.3)	(0.3)	(0.2)
Asylum seeker	0.0048	0.0092	0.016	-0.066	-0.061	0.083
	(0.1)	(0.1)	(0.1)	(0.1)	(0.1)	(0.1)
No doc. / waiting	0.12	-0.045	0.16	-0.17	-0.59**	-0.62**
	(0.2)	(0.2)	(0.2)	(0.2)	(0.3)	(0.3)
Denied protection	-2.36***	-1.71***	-2.47***	-1.13**	-1.10	-1.76***
	(0.6)	(0.5)	(0.9)	(0.4)	(0.8)	(0.3)
**Political attitude**						
Social dominance orientation	0.034**	0.027	0.013	0.011	0.058***	0.049***
	(0.02)	(0.02)	(0.02)	(0.02)	(0.02)	(0.02)
Right-wing authoritarianism	0.055***	0.080***	0.082***	0.099***	0.091***	0.064***
	(0.02)	(0.02)	(0.03)	(0.03)	(0.02)	(0.02)
Constant	3.00***	2.44***	1.92***	1.98***	2.08***	2.06***
	(0.4)	(0.5)	(0.6)	(0.6)	(0.6)	(0.6)
Observations	676	675	675	677	675	675
R^2	0.162	0.150	0.212	0.248	0.242	0.269

OLS regressions, robust standard errors in parentheses

* p<0.10, ** p<0.05, *** p<0.01

Dependent variables: Self-reported trust in institutions in Turkey, 1 = “Do not trust at all”, 2 = “Do not trust very much”, 3 = “I don’t know”, 4 = “Trust somewhat”, 5 = “Trust completely”

Independent variables:

Exposure: Additive index of the 16-item Harvard Trauma Questionnaire

Female: dummy variable indicating respondent's gender; 0 = male, 1 = female

Age: proxy of age; 1 = 18-24, 2 = 25-34, 3 = 35-44, 4 = 45-54, 5 = 55-64, 6 = 65-74, 7 = 75-84, 8 = 85 or older

Syria: dummy variable indicating respondent's country of origin; 0 = Iraq, 1 = Syria

Social / economic status: Self-reported status in country of origin, 0 (the worst off) … 10 (the best off)

Urban: Dummy variable indicating whether a respondent self-reported to come from a rural (0) or urban (1) region

Arrived in 2016: Dummy variable indicating whether a respondent arrived in Turkey in 2016 (1) or not (0) region

Further controls:

Education: Indicator variable, 1 = no formal education, 2 = < 6 years of schooling, 3 = 6 years of schooling, 4 = 9 years of schooling, 5 = 12 years of schooling, 6 = > 12 years of schooling

Return: Indicator variable on belief to move back; 0 = No, I do not think I will move back; 1 = Yes, 0 to 4 years from now; 2 = Yes, 5 to 10 years from now; 3 = Yes, when I am old.
Legal status: Indicator variable on current legal status in Turkey, 1 = Turkish citizen, 1 = Permanent protection, 3 = Temporary protection, 4 = Temporary suspension of deportation, 5 = Asylum seeker, 6 = No documentation or waiting for documentation, 7 = Denied protection, 8 = Other
Social dominance orientation: Additive index of 6 items of Social Dominance Orientation Scale

Right-wing authoritarianism: Additive index of 3 items of Right-Wing Authoritarianism Scale

Supplementary Table 11: Exposure to Violence and Institutional Trust at a level of Subsamples: Indiscriminate shelling or bombing

	(1)	(2)	(3)	(4)	(5)	(6)
	Courts	Police	Politicians	Parties	Parliament	Government
Exposure to violence	0.037***	0.047***	-0.022	-0.037**	-0.078***	-0.076***
	(0.01)	(0.01)	(0.02)	(0.02)	(0.01)	(0.01)
**Demographics**						
female	-0.15*	-0.11	-0.079	-0.042	-0.38***	-0.34***
	(0.09)	(0.09)	(0.1)	(0.1)	(0.1)	(0.10)
age	0.018	-0.019	-0.0091	-0.081**	0.025	0.028
	(0.03)	(0.03)	(0.04)	(0.04)	(0.04)	(0.03)
Syria	-0.32***	-0.067	-0.52***	-0.47***	-0.20	-0.078
	(0.1)	(0.1)	(0.1)	(0.1)	(0.1)	(0.1)
Social / economic status	-0.053***	-0.060***	0.00014	-0.051**	-0.079***	-0.097***
	(0.02)	(0.02)	(0.02)	(0.02)	(0.02)	(0.02)
**Education**						
< 6 years of schooling	0.21	-0.063	0.15	0.074	0.024	0.86
	(0.3)	(0.5)	(0.5)	(0.5)	(0.6)	(0.6)
6 years of schooling	-0.022	0.094	0.89**	0.82**	0.23	0.53
	(0.3)	(0.4)	(0.4)	(0.4)	(0.4)	(0.5)
9 years of schooling	0.17	0.69*	1.14***	1.29***	0.56	1.01**
	(0.3)	(0.4)	(0.3)	(0.3)	(0.4)	(0.5)
12 years of schooling	0.057	0.55	1.00***	0.94***	0.36	0.83*
	(0.3)	(0.4)	(0.3)	(0.3)	(0.4)	(0.5)
> 12 years of schooling	-0.16	0.29	0.82**	0.87***	0.21	0.63
	(0.3)	(0.4)	(0.3)	(0.3)	(0.4)	(0.5)
urban	0.025	0.073	0.010	-0.10	0.038	0.060
	(0.1)	(0.1)	(0.2)	(0.2)	(0.2)	(0.2)
Arrived in 2016	0.45***	0.21*	0.10	0.067	0.22	0.50***
	(0.1)	(0.1)	(0.2)	(0.2)	(0.1)	(0.1)
**Prospects of moving back**						
In 0-4 years	0.23*	0.24*	0.69***	0.76***	0.58***	0.47***

	(0.1)	(0.1)	(0.2)	(0.2)	(0.1)	(0.1)
In 5-10 years	0.064	0.13	0.16	0.25*	0.28**	0.23*
	(0.1)	(0.1)	(0.1)	(0.1)	(0.1)	(0.1)
When I am old.	0.27*	-0.035	-0.64***	-0.61***	0.096	0.29
	(0.2)	(0.2)	(0.2)	(0.2)	(0.2)	(0.2)
Constant	4.25***	3.80***	2.74***	3.11***	4.06***	3.79***
	(0.3)	(0.4)	(0.4)	(0.4)	(0.4)	(0.5)
Observations	529	525	527	528	530	528
R^2	0.112	0.115	0.163	0.205	0.223	0.237

OLS regressions, robust standard errors in parentheses

* p<0.10, ** p<0.05, *** p<0.01

Sample restricted to those who reported to have experienced indiscriminate shelling or bombing

Dependent variables: Self-reported trust in institutions in Turkey, 1 = "Do not trust at all", 2 = "Do not trust very much", 3 = "I don't know", 4 = "Trust somewhat", 5 = "Trust completely"

Independent variables:

Exposure: Additive index of the 16-item Harvard Trauma Questionnaire

Female: dummy variable indicating respondent's gender; 0 = male, 1 = female

Age: proxy of age; 1 = 18-24, 2 = 25-34, 3 = 35-44, 4 = 45-54, 5 = 55-64, 6 = 65-74, 7 = 75-84, 8 = 85 or older

Syria: dummy variable indicating respondent's country of origin; 0 = Iraq, 1 = Syria

Further controls:

Social / economic status: Self-reported status in country of origin, 0 (the worst off) … 10 (the best off)

Education: Indicator variable, 1 = no formal education, 2 = < 6 years of schooling, 3 = 6 years of schooling, 4 = 9 years of schooling, 5 = 12 years of schooling, 6 = > 12 years of schooling

Urban: Dummy variable indicating whether a respondent self-reported to come from a rural (0) or urban (1) region

Return: Indicator variable on belief to move back; 0 = No, I do not think I will move back; 1 = Yes, 0 to 4 years from now; 2 = Yes, 5 to 10 years from now; 3 = Yes, when I am old.

Supplementary Table 12: Exposure to Violence and Institutional Trust at a level of Subsamples: Forced evacuation

	(1) Courts	(2) Police	(3) Politicians	(4) Parties	(5) Parliament	(6) Government
Exposure to violence	0.0027	0.028**	-0.036*	-0.054***	-0.10***	-0.12***
	(0.01)	(0.01)	(0.02)	(0.02)	(0.02)	(0.02)
**Demographics**						
female	-0.15	-0.082	-0.18	-0.10	-0.38***	-0.37***
	(0.09)	(0.1)	(0.1)	(0.1)	(0.1)	(0.1)
age	0.041	-0.0035	-0.012	-0.039	0.026	0.049
	(0.03)	(0.04)	(0.05)	(0.05)	(0.05)	(0.04)
Syria	-0.29**	0.00036	-0.66***	-0.56***	-0.28*	-0.059
	(0.1)	(0.1)	(0.2)	(0.2)	(0.2)	(0.1)
Social / economic status	-0.036**	-0.055***	-0.021	-0.073***	-0.089***	-0.087***
	(0.02)	(0.02)	(0.03)	(0.03)	(0.02)	(0.02)
**Education**						
< 6 years of schooling	0.90***	0.0097	-0.33	-0.0084	0.33	0.24
	(0.3)	(0.6)	(0.9)	(0.8)	(0.6)	(0.9)
6 years of schooling	0.50*	0.18	-0.021	-0.23	-0.71	-0.056
	(0.3)	(0.3)	(0.6)	(0.6)	(0.4)	(0.4)
9 years of schooling	0.79***	0.69**	0.37	0.53	-0.10	0.44
	(0.2)	(0.3)	(0.5)	(0.6)	(0.4)	(0.4)
12 years of schooling	0.59***	0.49*	0.047	0.048	-0.34	0.18
	(0.2)	(0.3)	(0.5)	(0.6)	(0.4)	(0.3)
> 12 years of schooling	0.42*	0.22	-0.057	-0.11	-0.46	0.10
	(0.2)	(0.3)	(0.5)	(0.6)	(0.4)	(0.3)
urban	0.016	0.18	0.0071	-0.24	0.15	0.14
	(0.2)	(0.2)	(0.2)	(0.2)	(0.2)	(0.2)
Arrived in 2016	0.31**	0.025	0.37*	0.47**	0.20	0.50***
	(0.1)	(0.2)	(0.2)	(0.2)	(0.2)	(0.2)
**Prospects of moving back**						
In 0-4 years	0.11	0.16	0.69***	0.83***	0.48***	0.19

	(0.1)	(0.1)	(0.2)	(0.2)	(0.2)	(0.2)
In 5-10 years	-0.0017	0.13	0.23	0.37**	0.17	0.0060
	(0.1)	(0.1)	(0.2)	(0.2)	(0.1)	(0.1)
When I am old.	-0.22	-0.46*	-0.98***	-0.73***	-0.42	-0.29
	(0.2)	(0.2)	(0.3)	(0.3)	(0.3)	(0.2)
Constant	3.97***	3.93***	3.96***	4.26***	5.10***	4.85***
	(0.3)	(0.3)	(0.6)	(0.6)	(0.4)	(0.4)
Observations	385	384	384	384	384	383
R^2	0.117	0.127	0.237	0.280	0.315	0.312

OLS regressions, robust standard errors in parentheses

* p<0.10, ** p<0.05, *** p<0.01

Sample restricted to those who reported to have experienced forced evacuation

Dependent variables: Self-reported trust in institutions in Turkey, 1 = "Do not trust at all", 2 = "Do not trust very much", 3 = "I don't know", 4 = "Trust somewhat", 5 = "Trust completely"

Independent variables:

Exposure: Additive index of the 16-item Harvard Trauma Questionnaire

Female: dummy variable indicating respondent's gender; 0 = male, 1 = female

Age: proxy of age; 1 = 18-24, 2 = 25-34, 3 = 35-44, 4 = 45-54, 5 = 55-64, 6 = 65-74, 7 = 75-84, 8 = 85 or older

Syria: dummy variable indicating respondent's country of origin; 0 = Iraq, 1 = Syria

Further controls:

Social / economic status: Self-reported status in country of origin, 0 (the worst off) … 10 (the best off)

Education: Indicator variable, 1 = no formal education, 2 = < 6 years of schooling, 3 = 6 years of schooling, 4 = 9 years of schooling, 5 = 12 years of schooling, 6 = > 12 years of schooling

Urban: Dummy variable indicating whether a respondent self-reported to come from a rural (0) or urban (1) region

Return: Indicator variable on belief to move back; 0 = No, I do not think I will move back; 1 = Yes, 0 to 4 years from now; 2 = Yes, 5 to 10 years from now; 3 = Yes, when I am old.

Supplementary Table 13: Exposure to Violence and Institutional Trust, Ordered Logistic Regressions

	(1)	(2)	(3)	(4)	(5)	(6)
	Courts	Police	Politicians	Parties	Parliament	Government
exposure to violence	0.044**	0.082***	-0.024	-0.024	-0.11***	-0.12***
	(0.02)	(0.02)	(0.02)	(0.02)	(0.02)	(0.02)
**Demographics**						
female	-0.32**	-0.11	-0.0033	0.13	-0.41***	-0.45***
	(0.2)	(0.2)	(0.1)	(0.1)	(0.1)	(0.2)
age	0.070	-0.016	-0.023	-0.100*	0.070	0.062
	(0.06)	(0.06)	(0.05)	(0.05)	(0.06)	(0.06)
Syria	-0.78***	-0.19	-0.80***	-0.69***	-0.42**	-0.54***
	(0.2)	(0.2)	(0.2)	(0.2)	(0.2)	(0.2)
Social / economic status	-0.10***	-0.080***	-0.017	-0.098***	-0.14***	-0.18***
	(0.03)	(0.03)	(0.03)	(0.03)	(0.03)	(0.03)
urban	0.044	0.33	0.11	0.037	0.22	0.17
	(0.2)	(0.2)	(0.2)	(0.2)	(0.2)	(0.2)
Arrived in 2016	0.73***	0.17	0.18	0.12	0.28	0.78***
	(0.3)	(0.2)	(0.2)	(0.2)	(0.2)	(0.2)
**Education**						
< 6 years of schooling	0.56	-0.15	-0.37	-0.065	-0.20	1.37
	(0.6)	(0.5)	(0.7)	(0.6)	(0.7)	(0.8)
6 years of schooling	-0.065	0.40	0.67	0.65	0.063	0.53
	(0.5)	(0.4)	(0.4)	(0.4)	(0.5)	(0.6)
9 years of schooling	0.61	1.58***	1.40***	1.53***	0.83*	1.53***
	(0.4)	(0.4)	(0.4)	(0.4)	(0.5)	(0.5)
12 years of schooling	0.45	1.19***	0.99**	0.93**	0.37	1.16**
	(0.4)	(0.4)	(0.4)	(0.4)	(0.5)	(0.5)
> 12 years of schooling	0.19	0.74**	0.65	0.70*	0.22	1.10**
	(0.4)	(0.4)	(0.4)	(0.4)	(0.4)	(0.5)
**Prospects of moving back**						
0 to 4 years from now.	0.62***	0.62***	1.08***	1.25***	0.99***	0.80***
	(0.2)	(0.2)	(0.2)	(0.2)	(0.2)	(0.2)

Yes, 5 to 10 years from now.	0.46**	0.36*	0.36**	0.39**	0.52***	0.44**
	(0.2)	(0.2)	(0.2)	(0.2)	(0.2)	(0.2)
Yes, when I am old.	0.28	0.018	-0.69***	-0.63***	0.13	0.38
	(0.2)	(0.3)	(0.2)	(0.2)	(0.2)	(0.3)
cut1						
Constant	-3.67***	-2.53***	-1.73***	-2.10***	-3.81***	-3.39***
	(0.5)	(0.5)	(0.5)	(0.5)	(0.5)	(0.6)
cut2						
Constant	-2.42***	-1.02**	-0.31	-0.52	-1.88***	-1.83***
	(0.5)	(0.5)	(0.5)	(0.5)	(0.5)	(0.6)
cut3						
Constant	-1.31***	-0.37	0.47	0.32	-0.90*	-0.79
	(0.5)	(0.5)	(0.5)	(0.5)	(0.5)	(0.6)
cut4						
Constant	0.37	1.32***	1.67***	1.44***	0.36	0.46
	(0.5)	(0.5)	(0.5)	(0.5)	(0.5)	(0.6)
Observations	713	710	709	711	712	710
Pseudo R^2	0.052	0.050	0.063	0.072	0.077	0.091

Ordered logistic regressions, robust standard errors in parentheses

* p<0.10, ** p<0.05, *** p<0.01

Dependent variables: Self-reported trust in institutions in Turkey, 1 = “Do not trust at all”, 2 = “Do not trust very much”, 3 = “I don’t know”, 4 = “Trust somewhat”, 5 = “Trust completely”

Independent variables:

Exposure: Additive index of the 16-item Harvard Trauma Questionnaire

Female: dummy variable indicating respondent’s gender; 0 = male, 1 = female

Age: proxy of age; 1 = 18-24, 2 = 25-34, 3 = 35-44, 4 = 45-54, 5 = 55-64, 6 = 65-74, 7 = 75-84, 8 = 85 or older

Syria: dummy variable indicating respondent’s country of origin; 0 = Iraq, 1 = Syria

Social / economic status: Self-reported status in country of origin, 0 (the worst off) … 10 (the best off)

Urban: Dummy variable indicating whether a respondent self-reported to come from a rural (0) or urban (1) region

Arrived in 2016: Dummy variable indicating whether a respondent arrived in Turkey in 2016 (1) or not (0) region

Further controls:

Education: Indicator variable, 1 = no formal education, 2 = < 6 years of schooling, 3 = 6 years of schooling, 4 = 9 years of schooling, 5 = 12 years of schooling, 6 = > 12 years of schooling

Return: Indicator variable on belief to move back; 0 = No, I do not think I will move back; 1 = Yes, 0 to 4 years from now; 2 = Yes, 5 to 10 years from now; 3 = Yes, when I am old.

Supplementary Table 14: The impact of subtypes of traumatic events on Trust (Table 5) with all coefficients

	(1) Social Trust	(2) Courts	(3) Police	(4) Politicians	(5) Parties	(6) Parliament	(7) Government
Personal trauma self / others	0.31***	-0.013	-0.024	-0.15***	-0.22***	-0.29***	-0.26***
	(0.06)	(0.02)	(0.02)	(0.03)	(0.03)	(0.03)	(0.03)
Kidnapping & torture	0.016	-0.039	0.018	-0.047	0.019	-0.16**	-0.065
	(0.2)	(0.07)	(0.08)	(0.09)	(0.09)	(0.08)	(0.09)
Forced evacuation, near death	-0.17	0.14**	0.13**	0.14*	0.22***	0.22***	0.22***
	(0.1)	(0.06)	(0.06)	(0.07)	(0.07)	(0.06)	(0.06)
Siege	-0.026	0.061**	0.12***	0.12***	0.16***	0.13***	0.069**
	(0.07)	(0.03)	(0.03)	(0.04)	(0.04)	(0.03)	(0.03)
**Demographics**							
Female	-0.12	-0.15**	-0.074	-0.029	0.020	-0.31***	-0.28***
	(0.2)	(0.08)	(0.08)	(0.10)	(0.09)	(0.08)	(0.08)
Age	0.064	0.025	-0.020	-0.034	-0.092***	0.0099	0.023
	(0.06)	(0.03)	(0.03)	(0.04)	(0.03)	(0.03)	(0.03)
Syria	-0.69***	-0.32***	-0.048	-0.49***	-0.35***	-0.056	-0.090
	(0.2)	(0.09)	(0.10)	(0.1)	(0.1)	(0.1)	(0.1)
Social / economic status	-0.057	-0.041***	-0.026	0.023	-0.028	-0.036**	-0.059***
	(0.04)	(0.01)	(0.02)	(0.02)	(0.02)	(0.02)	(0.02)
**Education**							
< 6 years of schooling	0.58	0.18	-0.17	-0.24	-0.14	-0.29	0.76
	(0.9)	(0.3)	(0.4)	(0.4)	(0.4)	(0.4)	(0.5)
6 years of schooling	-0.37	-0.16	0.23	0.50	0.42	-0.079	0.31
	(0.8)	(0.2)	(0.3)	(0.3)	(0.3)	(0.3)	(0.4)
9 years of schooling	-0.18	0.15	0.72***	0.86***	0.96***	0.28	0.81**
	(0.7)	(0.2)	(0.3)	(0.3)	(0.3)	(0.3)	(0.3)

12 years of schooling	0.090	0.067	0.62**	0.67**	0.58**	0.079	0.68**
	(0.7)	(0.2)	(0.3)	(0.3)	(0.3)	(0.3)	(0.3)
> 12 years of schooling	-0.25	-0.014	0.45*	0.48*	0.51**	0.073	0.68**
	(0.7)	(0.2)	(0.3)	(0.3)	(0.2)	(0.3)	(0.3)
Urban	-0.045	-0.0077	0.095	0.025	0.026	0.16	0.11
	(0.3)	(0.1)	(0.1)	(0.1)	(0.1)	(0.1)	(0.1)
Arrived in 2016	0.73***	0.29***	0.046	0.13	0.14	0.14	0.47***
	(0.2)	(0.1)	(0.1)	(0.1)	(0.1)	(0.1)	(0.10)
**Prospects of moving back**							
Yes, 0 to 4 years from now.	0.75***	0.18*	0.14	0.54***	0.58***	0.26**	0.19*
	(0.3)	(0.1)	(0.1)	(0.1)	(0.1)	(0.1)	(0.1)
Yes, 5 to 10 years from now.	0.32	0.14	0.079	0.16	0.14	0.17	0.15
	(0.2)	(0.10)	(0.1)	(0.1)	(0.1)	(0.1)	(0.1)
Yes, when I am old.	0.58*	0.11	-0.023	-0.60***	-0.62***	-0.054	0.10
	(0.3)	(0.1)	(0.1)	(0.2)	(0.2)	(0.2)	(0.1)
Constant	-1.16	4.12***	3.48***	2.80***	2.84***	3.68***	3.43***
	(0.7)	(0.2)	(0.3)	(0.3)	(0.3)	(0.3)	(0.4)
Observations	726	713	710	709	711	712	710
(Pseudo)R^2	0.070	0.107	0.106	0.198	0.259	0.276	0.277

(1): Logistic regression, (2)-(7): OLS, robust standard errors in parentheses

* p<0.10, ** p<0.05, *** p<0.01

Dependent variables:
(1): Social Trust (binary measure, 0 = “most cannot be trusted” / “can't be too careful” / “it depends” / “I don’t know”; 1 = “most people can be trusted”)

(2)-(7): Self-reported trust in institutions in Turkey, 1 = “Do not trust at all”, 2 = “Do not trust very much”, 3 = “I don’t know”, 4 = “Trust somewhat”, 5 = “Trust completely”

Independent variables:

Four types of conflict experiences derived by PCA:

Personal Trauma to Self and Others: Includes having experienced Serious injury, Combat situation, Forced separation from family, Murder of family member or friend, Unnatural death of family member or friend and Murder of stranger or strangers

Kidnapping and Torture: having been kidnapped or tortured

Forced Evacuation and Close to Death: Having been forcibly evacuated or close to death

Siege: includes Lack of food or water, Ill health without medical care, Lack of shelter, Indiscriminate shelling or bombing.

Demographic controls:

Female: dummy variable indicating respondent's gender; 0 = male, 1 = female

Age: proxy of age; 1 = 18-24, 2 = 25-34, 3 = 35-44, 4 = 45-54, 5 = 55-64, 6 = 65-74, 7 = 75-84, 8 = 85 or older

Syria: dummy variable indicating respondent's country of origin; 0 = Iraq, 1 = Syria

Social / economic status: Self-reported status in country of origin, 0 (the worst off) … 10 (the best off)

Urban: Dummy variable indicating whether a respondent self-reported to come from a rural (0) or urban (1) region

Arrived in 2016: Dummy variable indicating whether a respondent arrived in Turkey in 2016 (1) or not (0) region

Further controls:

Education: Indicator variable, 1 = no formal education, 2 = < 6 years of schooling, 3 = 6 years of schooling, 4 = 9 years of schooling, 5 = 12 years of schooling, 6 = > 12 years of schooling

Return: Indicator variable on belief to move back; 0 = No, I do not think I will move back; 1 = Yes, 0 to 4 years from now; 2 = Yes, 5 to 10 years from now; 3 = Yes, when I am old.

Supplementary Table 15: The impact of Symptoms of Posttraumatic Stress on Trust (Table 6) with all coefficients

	(1)<br>Social Trust	(2)<br>Courts	(3)<br>Police	(4)<br>Politicians	(5)<br>Parties	(6)<br>Parliament	(7)<br>Government
ptsd	0.011	-0.025***	-0.013*	-0.026***	-0.019**	-0.054***	-0.064***
	(0.02)	(0.007)	(0.007)	(0.009)	(0.009)	(0.008)	(0.007)
**Demographics**							
female	-0.12	-0.15**	-0.049	0.011	0.070	-0.22**	-0.22***
	(0.2)	(0.08)	(0.08)	(0.10)	(0.10)	(0.09)	(0.08)
age	0.066	0.042	-0.00024	-0.016	-0.074**	0.028	0.037
	(0.06)	(0.03)	(0.03)	(0.04)	(0.04)	(0.03)	(0.03)
Syria	-0.52**	-0.34***	-0.12	-0.59***	-0.49***	-0.25**	-0.22**
	(0.2)	(0.09)	(0.10)	(0.1)	(0.1)	(0.1)	(0.10)
Social / economic status	0.0013	-0.042***	-0.027*	0.0028	-0.064***	-0.073***	-0.091***
	(0.03)	(0.01)	(0.02)	(0.02)	(0.02)	(0.02)	(0.02)
urban	-0.11	-0.063	0.039	0.020	0.026	0.13	0.11
	(0.3)	(0.1)	(0.1)	(0.1)	(0.1)	(0.1)	(0.1)
Arrived in 2016	0.75***	0.29***	0.023	0.12	0.11	0.046	0.40***
	(0.2)	(0.1)	(0.1)	(0.1)	(0.1)	(0.1)	(0.1)
**Education**							
< 6 years of schooling	0.61	0.37	-0.18	-0.33	-0.22	-0.22	0.82*
	(0.9)	(0.3)	(0.4)	(0.5)	(0.4)	(0.5)	(0.5)
6 years of schooling	-0.50	0.072	0.42	0.67**	0.59**	0.048	0.37
	(0.8)	(0.2)	(0.3)	(0.3)	(0.3)	(0.3)	(0.4)
9 years of schooling	-0.031	0.36*	0.91***	0.97***	1.03***	0.28	0.84**
	(0.7)	(0.2)	(0.3)	(0.3)	(0.3)	(0.3)	(0.4)
12 years of schooling	0.30	0.28	0.80***	0.71**	0.65**	0.033	0.66*
	(0.6)	(0.2)	(0.3)	(0.3)	(0.3)	(0.3)	(0.4)

> 12 years of schooling	0.075	0.18	0.60**	0.47	0.49*	-0.012	0.62*
	(0.6)	(0.2)	(0.3)	(0.3)	(0.3)	(0.3)	(0.4)
**Prospects of moving back**							
In 0-4 years	0.18	0.077	0.11	0.66***	0.80***	0.51***	0.33***
	(0.2)	(0.1)	(0.1)	(0.1)	(0.1)	(0.1)	(0.1)
In 5-10 years	-0.037	0.065	0.021	0.21*	0.25**	0.30***	0.23**
	(0.2)	(0.10)	(0.1)	(0.1)	(0.1)	(0.1)	(0.1)
When I am old.	-0.044	-0.082	-0.21	-0.61***	-0.54***	0.082	0.15
	(0.3)	(0.1)	(0.1)	(0.2)	(0.2)	(0.2)	(0.1)
Constant	-1.20	4.65***	3.93***	3.37***	3.38***	4.69***	4.55***
	(0.7)	(0.3)	(0.4)	(0.4)	(0.4)	(0.4)	(0.4)
Observations	700	691	689	689	691	690	690
(Pseudo) R^2	0.030	0.110	0.076	0.195	0.215	0.194	0.246

(1): Logistic regression, (2)-(7): OLS, robust standard errors in parentheses

* p<0.10, ** p<0.05, *** p<0.01

Dependent variables:
(1): Social Trust (binary measure, 0 = "most cannot be trusted" / "can't be too careful" / "it depends" / "I don't know"; 1 = "most people can be trusted")

(2)-(7): Self-reported trust in institutions in Turkey, 1 = "Do not trust at all", 2 = "Do not trust very much", 3 = "I don't know", 4 = "Trust somewhat", 5 = "Trust completely"

Independent variables:

PTSD: Additive index of 6 symptoms of posttraumatic stress according to PCL-C, for each 1 = "Not at all", 2 = "A little bit", 3 = "Moderately", 4 = "Quite a bit", 5 = "Extremely".

Demographic controls:

Female: dummy variable indicating respondent's gender; 0 = male, 1 = female

Age: proxy of age; 1 = 18-24, 2 = 25-34, 3 = 35-44, 4 = 45-54, 5 = 55-64, 6 = 65-74, 7 = 75-84, 8 = 85 or older

Syria: dummy variable indicating respondent's country of origin; 0 = Iraq, 1 = Syria

Social / economic status: Self-reported status in country of origin, 0 (the worst off) … 10 (the best off)

Urban: Dummy variable indicating whether a respondent self-reported to come from a rural (0) or urban (1) region

Arrived in 2016: Dummy variable indicating whether a respondent arrived in Turkey in 2016 (1) or not (0) region

Further controls:

Education: Indicator variable, 1 = no formal education, 2 = < 6 years of schooling, 3 = 6 years of schooling, 4 = 9 years of schooling, 5 = 12 years of schooling, 6 = > 12 years of schooling

Return: Indicator variable on belief to move back; 0 = No, I do not think I will move back; 1 = Yes, 0 to 4 years from now; 2 = Yes, 5 to 10 years from now; 3 = Yes, when I am old.

Supplementary Table 16: The impact of Posttraumatic Growth on Trust

	(1) Social Trust	(2) Courts	(3) Police	(4) Politicians	(5) Parties	(6) Parliament	(7) Government
ptg	-0.0073	-0.0052	0.0065	0.012*	0.026***	-0.00018	-0.0069
	(0.01)	(0.005)	(0.005)	(0.006)	(0.006)	(0.006)	(0.005)
**Demographics**							
female	-0.15	-0.14*	-0.076	0.014	0.072	-0.21**	-0.20**
	(0.2)	(0.08)	(0.08)	(0.10)	(0.10)	(0.09)	(0.09)
age	0.078	0.037	0.0050	-0.026	-0.088**	0.013	0.015
	(0.06)	(0.03)	(0.03)	(0.04)	(0.04)	(0.03)	(0.03)
Syria	-0.53**	-0.35***	-0.12	-0.60***	-0.53***	-0.25**	-0.26**
	(0.2)	(0.09)	(0.1)	(0.1)	(0.1)	(0.1)	(0.1)
Social / economic status	0.012	-0.046***	-0.036**	-0.011	-0.080***	-0.089***	-0.10***
	(0.03)	(0.01)	(0.02)	(0.02)	(0.02)	(0.02)	(0.02)
urban	-0.15	-0.051	0.059	0.088	0.082	0.24*	0.16
	(0.3)	(0.1)	(0.1)	(0.1)	(0.1)	(0.1)	(0.1)
arrived_2016	0.74***	0.26**	0.040	0.17	0.20	0.073	0.37***
	(0.2)	(0.1)	(0.1)	(0.1)	(0.1)	(0.1)	(0.1)
**Education**							
< 6 years of schooling	0.58	0.29	-0.26	-0.45	-0.43	-0.31	0.74
	(0.9)	(0.3)	(0.4)	(0.4)	(0.4)	(0.5)	(0.5)
6 years of schooling	-0.43	0.18	0.25	0.46	0.26	-0.059	0.42
	(0.8)	(0.2)	(0.3)	(0.3)	(0.3)	(0.3)	(0.4)
9 years of schooling	0.061	0.40**	0.84***	0.81***	0.74**	0.21	0.86**
	(0.7)	(0.2)	(0.3)	(0.3)	(0.3)	(0.3)	(0.4)
12 years of schooling	0.42	0.31	0.70**	0.51*	0.29	-0.091	0.61*
	(0.7)	(0.2)	(0.3)	(0.3)	(0.3)	(0.3)	(0.4)
> 12 years of	0.19	0.22	0.49*	0.24	0.092	-0.13	0.60*

schooling							
	(0.7)	(0.2)	(0.3)	(0.3)	(0.3)	(0.3)	(0.3)
**Prospects of moving back**							
In 0-4 years	0.23	0.19*	0.092	0.65***	0.69***	0.67***	0.57***
	(0.2)	(0.1)	(0.1)	(0.1)	(0.1)	(0.1)	(0.1)
In 5-10 years	-0.019	0.15	0.041	0.23*	0.23*	0.40***	0.36***
	(0.2)	(0.09)	(0.10)	(0.1)	(0.1)	(0.1)	(0.1)
When I am old.	-0.061	0.030	-0.15	-0.48***	-0.43***	0.31**	0.42***
	(0.3)	(0.1)	(0.1)	(0.2)	(0.2)	(0.2)	(0.1)
Constant	-0.89	4.37***	3.54***	2.64***	2.42***	3.80***	3.71***
	(0.7)	(0.3)	(0.4)	(0.4)	(0.4)	(0.3)	(0.4)
Observations	700	692	691	690	692	691	691
(Pseudo) R^2	0.032	0.096	0.079	0.184	0.227	0.138	0.166

(1): Logistic regression, (2)-(7): OLS, robust standard errors in parentheses

* p<0.10, ** p<0.05, *** p<0.01

Dependent variables:
(1): Social Trust (binary measure, 0 = “most cannot be trusted” / “can't be too careful” / “it depends” / “I don’t know”; 1 = “most people can be trusted”)

(2)-(7): Self-reported trust in institutions in Turkey, 1 = “Do not trust at all”, 2 = “Do not trust very much”, 3 = “I don’t know”, 4 = “Trust somewhat”, 5 = “Trust completely”

Independent variables:

PTG: Additive index of 10 experiences of change of the Posttraumatic Growth Inventory-Short Form (PTGI-SF), for each 1 = “Not at all”, 2 = “A little bit”, 3 = “Moderately”, 4 = “Quite a bit”, 5 = “Extremely”.

Demographic controls:

Female: dummy variable indicating respondent’s gender; 0 = male, 1 = female

Age: proxy of age; 1 = 18-24, 2 = 25-34, 3 = 35-44, 4 = 45-54, 5 = 55-64, 6 = 65-74, 7 = 75-84, 8 = 85 or older

Syria: dummy variable indicating respondent’s country of origin; 0 = Iraq, 1 = Syria

Social / economic status: Self-reported status in country of origin, 0 (the worst off) … 10 (the best off)

Urban: Dummy variable indicating whether a respondent self-reported to come from a rural (0) or urban (1) region

Arrived in 2016: Dummy variable indicating whether a respondent arrived in Turkey in 2016 (1) or not (0) region

Further controls:

Education: Indicator variable, 1 = no formal education, 2 = < 6 years of schooling, 3 = 6 years of schooling, 4 = 9 years of schooling, 5 = 12 years of schooling, 6 = > 12 years of schooling

Return: Indicator variable on belief to move back; 0 = No, I do not think I will move back; 1 = Yes, 0 to 4 years from now; 2 = Yes, 5 to 10 years from now; 3 = Yes, when I am old.

Supplementary Table 17: Exposure to Violence and Institutional Trust for Syrian refugees

	(1) Courts	(2) Police	(3) Politicians	(4) Parties	(5) Parliament	(6) Government
exposure to violence	0.041***	0.048***	-0.0077	-0.0063	-0.043**	-0.028
	(0.02)	(0.02)	(0.02)	(0.02)	(0.02)	(0.02)
**Demographics**						
female	-0.064	0.15	0.16	0.32**	-0.15	-0.18
	(0.1)	(0.1)	(0.2)	(0.1)	(0.1)	(0.1)
age	0.076	0.069	0.010	-0.040	0.066	0.11**
	(0.05)	(0.05)	(0.06)	(0.06)	(0.07)	(0.06)
Social / economic status	-0.030	0.00098	0.039	0.021	-0.044	-0.058*
	(0.03)	(0.03)	(0.03)	(0.03)	(0.03)	(0.03)
urban	-0.11	-0.11	-0.14	-0.12	0.026	0.0066
	(0.1)	(0.1)	(0.2)	(0.2)	(0.2)	(0.1)
Arrived in 2016	0.36**	0.0029	-0.068	-0.23	-0.012	0.37**
	(0.2)	(0.2)	(0.2)	(0.2)	(0.2)	(0.2)
**Education**						
< 6 years of schooling	0.52	-0.020	0.084	0.049	0.18	0.74
	(0.4)	(0.5)	(0.5)	(0.5)	(0.6)	(0.6)
6 years of schooling	0.10	0.53	1.31***	0.72*	0.093	0.22
	(0.4)	(0.4)	(0.4)	(0.4)	(0.5)	(0.5)
9 years of schooling	0.12	0.44	0.77**	0.61*	-0.31	0.20
	(0.3)	(0.4)	(0.3)	(0.3)	(0.4)	(0.4)
12 years of schooling	0.22	0.72*	0.90***	0.46	-0.14	0.10
	(0.3)	(0.4)	(0.3)	(0.3)	(0.4)	(0.4)
> 12 years of schooling	0.096	0.65*	0.87***	0.56**	0.021	0.42
	(0.3)	(0.4)	(0.3)	(0.3)	(0.4)	(0.4)
**Prospects of moving back**						
0 to 4 years from now.	0.15	0.12	0.51**	0.55**	0.38*	0.26
	(0.2)	(0.2)	(0.2)	(0.2)	(0.2)	(0.2)
Yes, 5 to 10 years from now.	0.12	0.034	-0.14	-0.16	0.19	0.073
	(0.2)	(0.2)	(0.2)	(0.2)	(0.2)	(0.2)

Yes, when I am old.	0.073	0.0078	-0.64***	-0.43*	0.19	0.097
	(0.2)	(0.2)	(0.2)	(0.2)	(0.2)	(0.2)
Constant	3.51***	3.00***	2.01***	2.31***	3.62***	3.38***
	(0.4)	(0.5)	(0.5)	(0.4)	(0.5)	(0.5)
Observations	286	284	283	285	283	284
R^2	0.085	0.069	0.115	0.109	0.083	0.091

OLS regressions, robust standard errors in parentheses

* p<0.10, ** p<0.05, *** p<0.01

Dependent variables: Self-reported trust in institutions in Turkey, 1 = "Do not trust at all", 2 = "Do not trust very much", 3 = "I don't know", 4 = "Trust somewhat", 5 = "Trust completely"

Independent variables:

Exposure: Additive index of the 16-item Harvard Trauma Questionnaire

Female: dummy variable indicating respondent's gender; 0 = male, 1 = female

Age: proxy of age; 1 = 18-24, 2 = 25-34, 3 = 35-44, 4 = 45-54, 5 = 55-64, 6 = 65-74, 7 = 75-84, 8 = 85 or older

Syria: dummy variable indicating respondent's country of origin; 0 = Iraq, 1 = Syria

Social / economic status: Self-reported status in country of origin, 0 (the worst off) … 10 (the best off)

Urban: Dummy variable indicating whether a respondent self-reported to come from a rural (0) or urban (1) region

Arrived in 2016: Dummy variable indicating whether a respondent arrived in Turkey in 2016 (1) or not (0) region

Further controls:

Education: Indicator variable, 1 = no formal education, 2 = < 6 years of schooling, 3 = 6 years of schooling, 4 = 9 years of schooling, 5 = 12 years of schooling, 6 = > 12 years of schooling

Return: Indicator variable on belief to move back; 0 = No, I do not think I will move back; 1 = Yes, 0 to 4 years from now; 2 = Yes, 5 to 10 years from now; 3 = Yes, when I am old.

Supplementary Table 18: Exposure to Violence and Institutional Trust for Iraqi refugees

	(1)<br>Courts	(2)<br>Police	(3)<br>Politicians	(4)<br>Parties	(5)<br>Parliament	(6)<br>Government
exposure to violence	0.015	0.037***	-0.027	-0.032*	-0.091***	-0.11***
	(0.01)	(0.01)	(0.02)	(0.02)	(0.02)	(0.01)
**Demographics**						
female	-0.17*	-0.19*	-0.12	-0.12	-0.32***	-0.28***
	(0.09)	(0.1)	(0.1)	(0.1)	(0.1)	(0.1)
age	0.0051	-0.039	-0.014	-0.063	0.031	-0.0047
	(0.03)	(0.03)	(0.04)	(0.04)	(0.04)	(0.03)
Social / economic status	-0.051***	-0.052***	-0.019	-0.10***	-0.086***	-0.10***
	(0.02)	(0.02)	(0.02)	(0.02)	(0.02)	(0.02)
urban	0.43	0.55**	0.16	0.054	0.36	0.15
	(0.3)	(0.3)	(0.3)	(0.3)	(0.4)	(0.3)
Arrived in 2016	0.099	-0.047	0.15	0.28	0.12	0.36***
	(0.1)	(0.2)	(0.2)	(0.2)	(0.2)	(0.1)
**Education**						
< 6 years of schooling	-0.25	-0.32	-0.52	-0.16	-0.63	1.08*
	(0.4)	(0.5)	(0.7)	(0.6)	(0.7)	(0.6)
6 years of schooling	-0.31	0.14	0.015	0.50	0.094	0.95*
	(0.3)	(0.4)	(0.5)	(0.5)	(0.5)	(0.6)
9 years of schooling	0.094	0.82**	0.76	1.22***	0.73	1.68***
	(0.3)	(0.4)	(0.5)	(0.5)	(0.5)	(0.5)
12 years of schooling	-0.045	0.60	0.46	0.79*	0.44	1.64***
	(0.3)	(0.4)	(0.5)	(0.5)	(0.5)	(0.5)
> 12 years of schooling	-0.095	0.33	0.11	0.48	0.21	1.36***
	(0.3)	(0.4)	(0.5)	(0.5)	(0.4)	(0.5)
**Prospects of moving back**						
0 to 4 years from now.	0.34***	0.32**	0.76***	0.90***	0.59***	0.47***
	(0.1)	(0.1)	(0.2)	(0.2)	(0.1)	(0.1)
Yes, 5 to 10 years from now.	0.22*	0.17	0.42***	0.43***	0.33**	0.37***
	(0.1)	(0.1)	(0.2)	(0.2)	(0.1)	(0.1)

Yes, when I am old.	0.24	0.047	-0.51**	-0.68***	-0.073	0.35*
	(0.2)	(0.2)	(0.3)	(0.2)	(0.2)	(0.2)
Constant	4.01***	3.40***	3.21***	3.20***	3.73***	3.23***
	(0.3)	(0.5)	(0.5)	(0.5)	(0.5)	(0.6)
Observations	427	426	426	426	429	426
R^2	0.084	0.147	0.165	0.266	0.256	0.332

OLS regressions, robust standard errors in parentheses

* p<0.10, ** p<0.05, *** p<0.01

Dependent variables: Self-reported trust in institutions in Turkey, 1 = “Do not trust at all”, 2 = “Do not trust very much”, 3 = “I don’t know”, 4 = “Trust somewhat”, 5 = “Trust completely”

Independent variables:

Exposure: Additive index of the 16-item Harvard Trauma Questionnaire

Female: dummy variable indicating respondent’s gender; 0 = male, 1 = female

Age: proxy of age; 1 = 18-24, 2 = 25-34, 3 = 35-44, 4 = 45-54, 5 = 55-64, 6 = 65-74, 7 = 75-84, 8 = 85 or older

Syria: dummy variable indicating respondent’s country of origin; 0 = Iraq, 1 = Syria

Social / economic status: Self-reported status in country of origin, 0 (the worst off) … 10 (the best off)

Urban: Dummy variable indicating whether a respondent self-reported to come from a rural (0) or urban (1) region

Arrived in 2016: Dummy variable indicating whether a respondent arrived in Turkey in 2016 (1) or not (0) region

Further controls:

Education: Indicator variable, 1 = no formal education, 2 = < 6 years of schooling, 3 = 6 years of schooling, 4 = 9 years of schooling, 5 = 12 years of schooling, 6 = > 12 years of schooling

Return: Indicator variable on belief to move back; 0 = No, I do not think I will move back; 1 = Yes, 0 to 4 years from now; 2 = Yes, 5 to 10 years from now; 3 = Yes, when I am old.

4 Survey Items – Screenshots of the relevant variables (English translations)

The survey was generated using Qualtrics software, Version [November 2016] of Qualtrics. Copyright © [2022] Qualtrics. Qualtrics and all other Qualtrics product or service names are registered trademarks or trademarks of Qualtrics, Provo, UT, USA. https://www.qualtrics.com

4.1 Dependent variables

GENERALIZED SOCIAL TRUST

Generally speaking, would you say that most people can be trusted, or that you can't be too careful in dealing with people?

- Most people can be trusted.
- Can't be too careful.
- It depends.
- Most people cannot be trusted.
- I don't know.

TRUST IN INSTITUTIONS

How much do you personally trust and feel confidence in each of the following institutions in Turkey?

	Trust completely	Trust somewhat	Do not trust very much	Do not trust at all	I don't know
The courts	○	○	○	○	○
The police	○	○	○	○	○
Politicians	○	○	○	○	○
Political parties	○	○	○	○	○
The parliament	○	○	○	○	○
The national government	○	○	○	○	○

TRUST IN ETHNIC GROUPS

For each of the groups below, please indicate to what extent you think members of this group can be trusted.

	Most can be trusted	Can't be too careful	It depends	Most cannot be trusted	I don't know
Your family	○	○	○	○	○
People in your current neighborhood	○	○	○	○	○
Christians	○	○	○	○	○
Kurds	○	○	○	○	○
Shia Arab	○	○	○	○	○
Alawi	○	○	○	○	○
Sunni Arab	○	○	○	○	○
Turkoman	○	○	○	○	○

4.2 Key independent variables

EXPOSURE TO POTENTIALLY TRAUMATIC EVENTS

Please indicate whether you have experienced any of the following in your life before arriving in Turkey. Select all that apply.

- ☐ Lack of food or water
- ☐ Ill health without medical care
- ☐ Lack of shelter
- ☐ Imprisonment
- ☐ Physical abuse
- ☐ Serious injury
- ☐ Combat situation
- ☐ Indiscriminate shelling or bombing
- ☐ Being close to death
- ☐ Forced evacuation
- ☐ Forced separation from family
- ☐ Murder of family or friend
- ☐ Unnatural death of family or friend
- ☐ Murder of stranger or strangers
- ☐ Kidnapped
- ☐ Torture

POSTTRAUMATIC STRESS

Below is a list of problems and complaints that people sometimes have in response to stressful life experiences. Please indicate how much you have been bothered by each problem in the last month.

	Not at all	A little bit	Moderately	Quite a bit	Extremely
Repeated, disturbing memories, thoughts, or images of a stressful experience from the past?					
Feeling very upset when something reminded you of a stressful experience from the past?					
Avoid activities or situations because they remind you of a stressful experience from the past?					
Feeling distant or cut off from other people?					
Feeling irritable or having angry outbursts?					
Having difficulty concentrating?					

POSTTRAUMATIC GROWTH

For each of the statements below, please indicate the degree to which this change occurred in your life as a result of all that has happened.

	Not at all	To a very small degree	To a small degree	To a moderate degree	To a great degree	To a very great degree
I changed my priorities about what is important in life.						
I have a greater appreciation for the value of my own life.						
I am able to do better things with my life.						
I have a better understanding of spiritual matters.						
I have a greater sense of closeness with others.						
I have established a new path for my life.						
I know better that I can handle difficulties.						
I have a stronger religious faith.						
I discovered that I'm stronger than I thought I was.						
I learned a great deal about how wonderful people are.						

4.3 Control variables

GENDER

What is your gender?

- Male
- Female

AGE

What is your age (in years)?

- Under 18
- 18 - 24
- 25 - 34
- 35 - 44
- 45 - 54
- 55 - 64
- 65 - 74
- 75 - 84
- 85 or older

ETHNICITY

Ethnicity.

- Arab
- Armenian
- Assyrian
- Circassian
- Kurd
- Syriac
- Turkoman
- Yazidi
- Other

RELIGION

Religion.

- Muslim Sunni
- Muslim Shia
- Christian
- Druze
- Yazidi
- Other

COUNTRY OF ORIGIN

Country of origin.

- Syria
- Iraq

URBAN / RURAL BACKGROUND

Would you consider the place where you grew up rural or urban?

- Rural
- Urban

EDUCATION

What is the highest level of education that you have completed?

- I have no formal education
- I have completed less than 6 years of schooling
- I have completed 6 years of schooling
- I have completed 9 years of schooling
- I have completed 12 years of schooling
- I have completed more than 12 years of schooling

SELF-REPORTED SOCIOECONOMIC STATUS

Imagine the society in your country of origin (Syria or Iraq) as arranged on a scale like the one shown below, where the worst off socially and economically are on the left (0) and the best off are on the right (10).

Please move the slider to select the place where you feel you stood prior to the war.

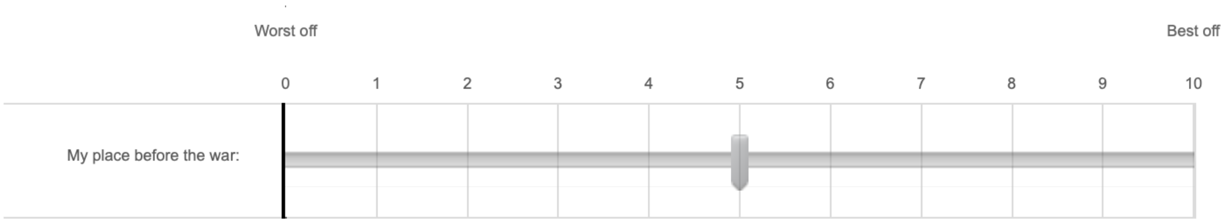

YEAR OF MIGRATION

When did you leave Syria or Iraq?

- ◯ 1969 or earlier
- ◯ 1970-1975
- ◯ 1976-1979
- ◯ 1980-1985
- ◯ 1986-1989
- ◯ 1990-1995
- ◯ 1996-1999
- ◯ 2000-2005
- ◯ 2006-2009
- ◯ 2010-2015
- ◯ 2016-today

YEAR OF ARRIVAL IN TURKEY

When did you arrive in Turkey?

- ◯ 1969 or earlier
- ◯ 1970-1975
- ◯ 1976-1979
- ◯ 1980-1985
- ◯ 1986-1989
- ◯ 1990-1995
- ◯ 1996-1999
- ◯ 2000-2005
- ◯ 2006-2009
- ◯ 2010-2015
- ◯ 2016-today

LEGAL STATUS

What is your current legal status in Turkey? Please select all that apply.

- ☐ Turkish citizen
- ☐ Permanent protection
- ☐ Temporary protection
- ☐ Temporary suspension of deportation
- ☐ Asylum seeker
- ☐ No documentation or waiting for documentation
- ☐ Denied protection
- ☐ Other

RETURN INTENTIONS

Do you think you will ever move back to your country of origin, or that of your parents, to live there permanently?

- ◯ Yes, 0 to 4 years from now.
- ◯ Yes, 5 to 10 years from now.
- ◯ Yes, when I am old.
- ◯ No, I do not think I will move back.

RIGHT-WING AUTHORITARIANISM

Please tell us whether you agree or disagree with the following statements:

	Strongly agree	Somewhat agree	Neither agree nor disagree	Somewhat disagree	Strongly disagree
Obedience and respect for authority are the most important virtues children should learn.	◯	◯	◯	◯	◯
Those who have rebelled against established religions are as virtuous as the devout.	◯	◯	◯	◯	◯
Young people should be allowed to challenge their parents' ways, confront established authorities, and in general, criticize the customs and traditions of our society	◯	◯	◯	◯	◯

SOCIAL DOMINANCE ORIENTATION

Please tell us whether you agree or disagree with the following statements:

	Strongly agree	Somewhat agree	Neither agree nor disagree	Somewhat disagree	Strongly disagree
It's probably a good thing that certain groups are at the top and other groups are at the bottom.	◯	◯	◯	◯	◯
Inferior groups should stay in their place.	◯	◯	◯	◯	◯
It would be good if all groups could be equal.	◯	◯	◯	◯	◯
Superior groups should dominate inferior groups.	◯	◯	◯	◯	◯
We should increase social equality.	◯	◯	◯	◯	◯
Group equality should be our ideal.	◯	◯	◯	◯	◯
